# The Genetic Architecture of Depression in Individuals of East Asian Ancestry: A Genome-Wide Association Study

**DOI:** 10.1001/jamapsychiatry.2021.2099

**Published:** 2021-09-29

**Authors:** Olga Giannakopoulou, Kuang Lin, Xiangrui Meng, Mei-Hsin Su, Po-Hsiu Kuo, Roseann E. Peterson, Swapnil Awasthi, Arden Moscati, Jonathan R. I. Coleman, Nick Bass, Iona Y. Millwood, Yiping Chen, Zhengming Chen, Hsi-Chung Chen, Mong-Liang Lu, Ming-Chyi Huang, Chun-Hsin Chen, Eli A. Stahl, Ruth J. F. Loos, Niamh Mullins, Robert J. Ursano, Ronald C. Kessler, Murray B. Stein, Srijan Sen, Laura J. Scott, Margit Burmeister, Yu Fang, Jess Tyrrell, Yunxuan Jiang, Chao Tian, Andrew M. McIntosh, Stephan Ripke, Erin C. Dunn, Kenneth S. Kendler, Robin G. Walters, Cathryn M. Lewis, Karoline Kuchenbaecker

**Affiliations:** 1 Division of Psychiatry, University College of London, London, United Kingdom; 2 Nuffield Department of Population Health, University of Oxford, Oxford, United Kingdom; 3 Institute of Epidemiology and Preventive Medicine, National Taiwan University College of Public Health, Taipei, Taiwan; 4 Department of Psychiatry, National Taiwan University Hospital, Taipei, Taiwan; 5 Virginia Institute for Psychiatric and Behavioral Genetics, Department of Psychiatry, Virginia Commonwealth University, Richmond, Virginia; 6 Department of Psychiatry and Psychotherapy, Charité - Universitätsmedizin, Berlin, Germany; 7 The Charles Bronfman Institute for Personalized Medicine, Icahn School of Medicine at Mount Sinai, New York, New York; 8 Social, Genetic, and Developmental Psychiatry Centre, Institute of Psychiatry, Psychology, and Neuroscience, King’s College London, London, United Kingdom; 9 National Institute for Health Research Maudsley Biomedical Research Centre, King’s College London, London, United Kingdom; 10 MRC Population Health Research Unit, University of Oxford, Oxford, United Kingdom; 11 Department of Psychiatry, Wan-Fang Hospital, Taipei, Taiwan; 12 School of Medicine, College of Medicine, Taipei Medical University, Taipei, Taiwan; 13 Department of Psychiatry, Taipei City Psychiatric Center, Taipei, Taiwan; 14 The Pamela Sklar Division of Psychiatric Genomics, Icahn School of Medicine at Mount Sinai, New York, New York; 15 The Mindich Child Health and Development Institute, Icahn School of Medicine at Mount Sinai, New York, New York; 16 Uniformed Services University of the Health Sciences, Bethesda, Maryland; 17 Harvard Medical School, Boston, Massachusetts; 18 University of California, San Diego, La Jolla, California; 19 Michigan Neuroscience Institute, Department of Psychiatry, University of Michigan, Ann Arbor, Michigan; 20 Department of Biostatistics, University of Michigan, Ann Arbor, Michigan; 21 Molecular & Behavioral Neuroscience Institute, Department of Computational Medicine & Bioinformatics, University of Michigan, Ann Arbor, Michigan; 22 Michigan Neuroscience Institute, University of Michigan, Ann Arbor, Michigan; 23 University of Exeter Medical School, University of Exeter, The RILD Building, RD&E Hospital, Exeter, United Kingdom; 24 23andme, Inc, Sunnyvale, California; 25 Division of Psychiatry, University of Edinburgh, Edinburgh, United Kingdom; 26 Analytic and Translational Genetics Unit, Massachusetts General Hospital, Boston, Massachusetts; 27 Stanley Center for Psychiatric Research, Broad Institute of MIT and Harvard, Cambridge, Massachusetts; 28 Psychiatric and Neurodevelopmental Genetics Unit, Center for Genomic Medicine, Massachusetts General Hospital, Boston, Massachusetts; 29 UCL Genetics Institute, University College of London, London, United Kingdom

## Abstract

**Importance:**

Most previous genome-wide association studies (GWAS) of depression have used data from individuals of European descent. This limits the understanding of the underlying biology of depression and raises questions about the transferability of findings between populations.

**Objective:**

To investigate the genetics of depression among individuals of East Asian and European descent living in different geographic locations, and with different outcome definitions for depression.

**Design, Setting, and Participants:**

Genome-wide association analyses followed by meta-analysis, which included data from 9 cohort and case-control data sets comprising individuals with depression and control individuals of East Asian descent. This study was conducted between January 2019 and May 2021.

**Exposures:**

Associations of genetic variants with depression risk were assessed using generalized linear mixed models and logistic regression. The results were combined across studies using fixed-effects meta-analyses. These were subsequently also meta-analyzed with the largest published GWAS for depression among individuals of European descent. Additional meta-analyses were carried out separately by outcome definition (clinical depression vs symptom-based depression) and region (East Asian countries vs Western countries) for East Asian ancestry cohorts.

**Main Outcomes and Measures:**

Depression status was defined based on health records and self-report questionnaires.

**Results:**

There were a total of 194 548 study participants (approximate mean age, 51.3 years; 62.8% women). Participants included 15 771 individuals with depression and 178 777 control individuals of East Asian descent. Five novel associations were identified, including 1 in the meta-analysis for broad depression among those of East Asian descent: rs4656484 (β = −0.018, SE = 0.003, *P* = 4.43x10^−8^) at 1q24.1. Another locus at 7p21.2 was associated in a meta-analysis restricted to geographically East Asian studies (β = 0.028, SE = 0.005, *P* = 6.48x10^−9^ for rs10240457). The lead variants of these 2 novel loci were not associated with depression risk in European ancestry cohorts (β = −0.003, SE = 0.005, *P* = .53 for rs4656484 and β = −0.005, SE = 0.004, *P* = .28 for rs10240457). Only 11% of depression loci previously identified in individuals of European descent reached nominal significance levels in the individuals of East Asian descent. The transancestry genetic correlation between cohorts of East Asian and European descent for clinical depression was *r* = 0.413 (SE = 0.159). Clinical depression risk was negatively genetically correlated with body mass index in individuals of East Asian descent (*r* = −0.212, SE = 0.084), contrary to findings for individuals of European descent.

**Conclusions and Relevance:**

These results support caution against generalizing findings about depression risk factors across populations and highlight the need to increase the ancestral and geographic diversity of samples with consistent phenotyping.

## Key Points

**Question:**

Are the genetic risk factors for depression the same in individuals of East Asian and European descent?

**Findings:**

In this genome-wide association meta-analysis of depression in 194 548 individuals with East Asian ancestry, 2 novel genetic associations were identified, one of which is specific to individuals of East Asian descent living in East Asian countries. There was limited evidence for transferability with only 11% of depression loci previously identified in individuals of European descent reaching nominal significance levels in the individuals of East Asian descent.

**Meaning:**

Caution is advised against generalizing findings about genetic risk factors for depression beyond the studied population.

## Teaser

This genetic association study investigates the genetics of depression across multiple data sets of individuals of East Asian and European descent living in different countries and within different nongenetic cultural contexts.

## Introduction

Depression affects an estimated 300 million people^1^ and represents a leading cause of health-related disabilities. More than 80% of the global burden affects low- and middle-income countries.^2,3^ To date, 102 genetic variants have been associated with depression liability.^4,5,6,7^ However, most previous genetic studies have been conducted in European ancestry cohorts.^8^ Extending this work to other population groups can yield new biological insights pertinent to specific populations and facilitate improved genetic risk prediction across ancestry groups.^9,10^

The manifestation of depression varies. In China, the disorder traditionally associated with serious stress is neurasthenia, characterized by strong physical and psychological fatigue.^11^ Depression-like presentations are becoming more common in recent times.^12^ However, somatic symptoms tend to be emphasized over emotional and cognitive symptoms.^13^ Previous studies of US individuals of European descent have reported the absence of high-arousal positive emotions, such as excitement or enthusiasm, as a main feature of depression, while presentations in Chinese individuals emphasize the absence of low-arousal positive states, such as peacefulness.^14,15,16^ Consequently, different items on depression scales tend to be useful markers of depression across populations and ethnic groups,^17,18,19^ raising questions about what depression means and how best to assess it cross-culturally for research.

In this study, we have combined data from the China, Oxford, and Virginia Commonwealth University Experimental Research on Genetic Epidemiology (CONVERGE) consortium,^20^ China Kadoorie Biobank (CKB), and the Taiwan-Major Depressive Disorder (MDD) study, as well as studies conducted in the US and UK that included participants of East Asian ancestry, to carry out the first (to our knowledge) large GWAS meta-analysis of depression among 194 548 individuals with East Asian ancestry. We aimed to identify novel depression loci, assess the transferability of genetic risk factors between individuals of European and East Asian descent, characterize the genetic architecture associated with different depression definitions, and compare the findings between ancestry cohorts.

## Methods

### Participating Studies and Depression Definitions

This genome-wide association study was conducted between January 2019 and May 2021. We included data from CKB, CONVERGE, and the Taiwan-MDD study, as well as US- and UK-based cohorts with DNA samples of individuals of East Asian descent: 23andMe Inc, Women’s Health Initiative (WHI), Mount Sinai Bio*Me* Biobank, Intern Health Study (IHS), the Study to Assess Risk and Resilience in Servicemembers (Army-STARRS), and UK Biobank (UKB). The data for WHI presented in the current publication are based on the use of study data downloaded from the dbGaP website, under phs000200.v12.p3. Details about these cohorts and data sets are available in eTable 1 in Supplement 1 and eAppendix 1 in Supplement 2. All participants provided written informed consent, and each study obtained approval from local ethical review boards. Genotyping data were exported from China to the Oxford CKB International Coordinating Centre under Data Export Approvals 2014-13 and 2015-39 from the Office of Chinese Human Genetic Resource Administration. The CKB analyses were conducted under project 2018-0018 as approved by the CKB Research Committee. Details of each cohort have been previously described.^20,21,22,23,24,25,26,27,28,29,30^ This study followed the Strengthening the Reporting of Genetic Association Studies (STREGA) reporting guideline.

This investigation was based on data from individuals with East Asian ancestry as defined by the investigators based on genetic information. For each study, a principal component analysis was carried out based on the genetic similarity of pairs of individuals. Individuals that clustered around a reference group with confirmed East Asian ancestry were included in this analysis.

We used a range of measures to define depression, including structured clinical interviews, medical health care records, symptom questionnaires, and self-completed surveys in a broad discovery association analysis of 15 771 depression cases and 178 777 controls (eTable 1 in Supplement 1). We also split the sample to perform outcome-specific analyses based on clinical depression or symptom-based depression. For the analysis based on clinical depression, participants reporting lifetime symptoms that were likely to fulfill *DSM* criteria for MDD and individuals diagnosed with a depressive disorder based on medical records from primary and secondary health care were classified as having depression. In this analysis (8223 patients with depression and 85 370 control participants), we combined data from CONVERGE, Taiwan-MDD study, UKB, Army-STARRS, Bio*Me*, and CKB. The symptom-based depression analysis used short questionnaires to identify those with self-reported depression symptoms in general population cohorts, including the CKB (CIDI-trigger symptoms), WHI, and IHS (6124 individuals with depression, 73 095 control participants). We conducted additional association analyses in which cohorts were regrouped by region: cohorts in East Asian countries (12 027 individuals with depression and 83 727 control participants) vs cohorts with participants of East Asian descent in the US and UK studies (3744 individuals with depression, 95 050 control participants) (eTable 1 in Supplement 1).

### Genetic Association Analyses and Meta-analyses

Genotyping and quality control are described in eAppendix 1 and eTable 2 in Supplement 2. Single-nucleotide variant (SNV)–level associations with depression were assessed using logistic regression in the 23andMe, Taiwan-MDD study, Army-STARRS, UKB, WHI, and IHS cohorts. Linear-mixed models were used in the association analysis for CONVERGE (FastLMM, version 2.06.20130802)^31^ as well as CKB and Bio*Me* (SAIGE, version 0.36.1)^32^ to adjust for population structure and relatedness. We assessed an additive per-allele model. Unstandardized β estimates and standard errors (SEs) were calculated. Age, sex, principal components, and study-specific covariates (eg, study arm in WHI) were included as covariates.

We performed a *z*-score weighted meta-analysis using METAL, version 2011-03-25^33^ for 13 163 200 genetic variants (eFigure 1 in Supplement 2). For all meta-analyses, results were restricted to variants present in at least 2 studies. We also performed a *z*-score weighted meta-analysis combining results from our analysis of individuals of East Asian descent and the publicly available summary statistics from the largest published GWAS of participants of European descent.^7^

### Reproducibility of Established Depression Loci

We assessed whether the associations of 102 established depression loci from the largest published European ancestry GWAS^7^ were reproducible in samples from individuals with East Asian ancestry. We compared this to the absolute number of associations out of the 102 that we are powered to observe if the effect size estimates in individuals of East Asian ancestry are consistent with the effect size estimates from the European ancestry studies. For benchmarking, we also assessed the reproducibility of these established loci in ancestry-matched cohorts. We used independent European ancestry GWAS for depression with different sample sizes (Bio*Me*, BioVU, FinnGen, 23andMe).

### Heritability and Genetic Correlations

We estimated the SNV heritability (*h*^2^) using linkage disequilibrium score regression^33^ and bivariate genome-based restricted maximum likelihood (GREML) implemented in the GCTA software version 1.92^34^ for the 2 large Chinese data sets, CONVERGE and CKB (symptom-based definition). For this analysis we applied several prevalence estimates, ranging from 6.5%^35^ to 15%.^6^

We estimated transancestry genetic correlations between depression in cohorts of East Asian descent and European descent using POPCORN, version 1.0.^36^ We only present genetic correlation estimates where the standard error was less than 0.3. For clinical depression in individuals of European descent, we used the summary statistics from 45 396 individuals with a *DSM*-based diagnosis of major depressive disorder and 97 250 control participants included in the latest GWAS,^7^ excluding UKB and 23andMe. Additionally, we generated a symptom-based definition for individuals of European descent using the Patient Health Questionnaire 9 and a cutoff score of 10.^25,37,38^

## Results

### Genome-Wide Association Meta-analysis of Depression in Individuals of East Asian Descent

**Figure 1.**
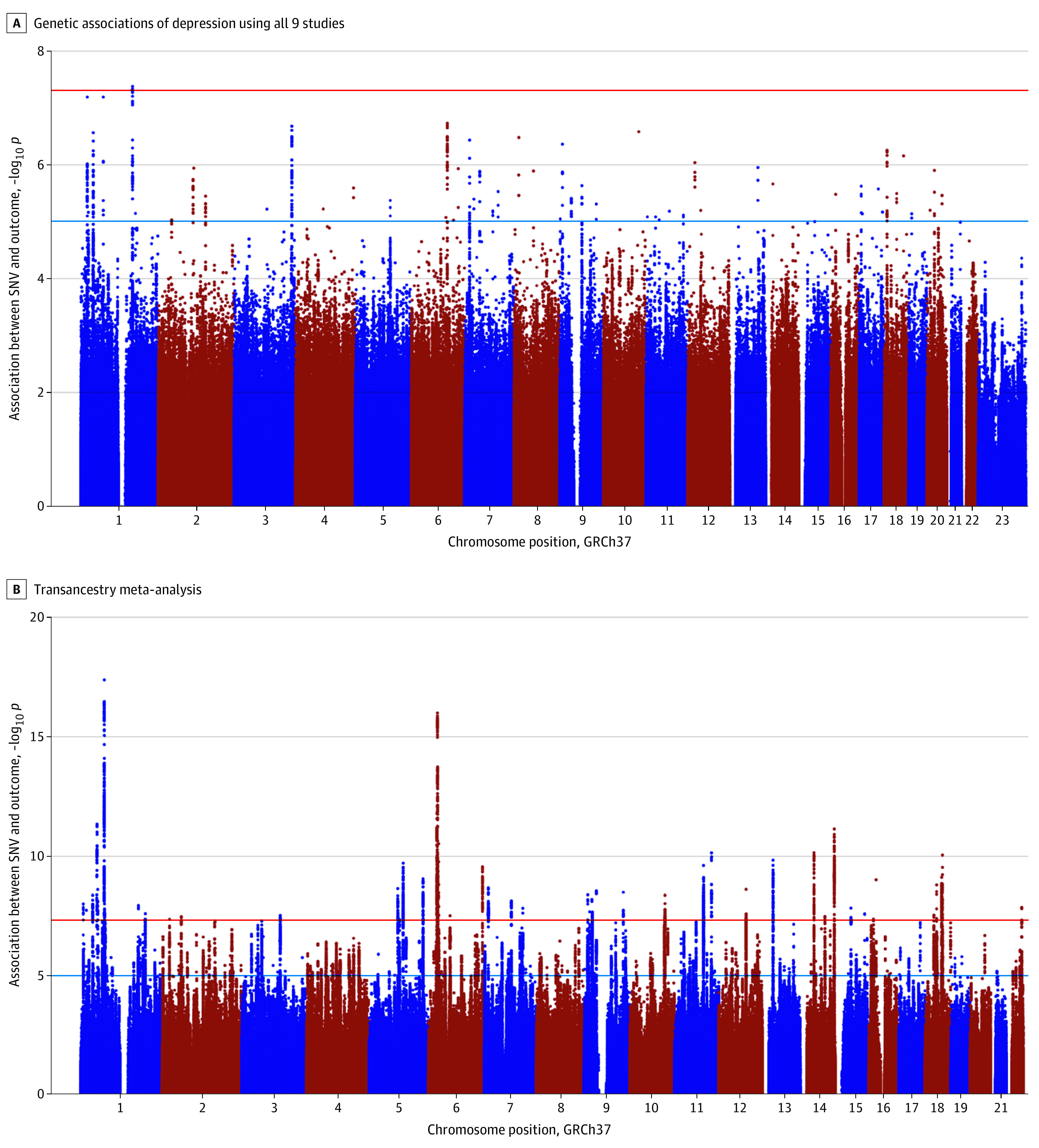
Manhattan Plot of the Genetic Associations With Depression in Ancestrally East Asian Samples Using All 9 Studies and the Transancestry Meta-analysis Between East Asian and European Ancestry Samples The y-axes show the −log_10_*P* values of the association between each single-nucleotide variant and the outcome. The x-axes show the chromosomal position (GRCh37). The red line represents the genome-wide significance threshold of 5 x 10^−8^ and the blue line, 10^−5^.

Participants included 15 771 individuals with depression and 178 777 control participants from 9 different studies^20,21,22,23,24,25,26,27,28,29,30,39,40^ (eTable 1 in Supplement 1). The meta-analysis yielded results for 9 223 944 variants with 1 region associated at genome-wide significance (Figure 1A; eTable 3 in Supplement 3). Variant rs4656484 at a previously unreported locus, 1q24.1, was associated with depression (β for C allele = −0.018, SE = 0.003, effect allele frequency [EAF] = 0.635, *P* = 4.4 × 10^−8^) (Table). It had consistent effect sizes across all studies except UKB (133 individuals with depression and 366 control participants) (eFigure 2 in Supplement 2). In the UK Brain Expression Consortium resource (UKBEC),^41^ rs4656484 was associated with expression of *LMX1A* (OMIM 600298), which has been linked to dopamine neuron development.^42^ The tissue group showing the strongest eQTL association was frontal cortex (*P* = 1.1 × 10^−4^).^42^

**Table.** Association Results With Depression for Novel Loci With *P* < 5 x 10^−8^ Based on Fixed-Effects Meta-analyses

rs-id^a^	CHR:position	EA/OA	Cohort	No. of individuals with depression; No. of control participants	EAF	β (SE)	OR (95% CI)^b^	*P* value
**Discovery set: East Asian ancestry GWAS of broad depression**								
rs4656484	1:166145466	C/G	EAS^c^	15 771; 178 777	0.63	−0.018 (0.003)	0.94 (0.91-0.97)	4.43×10^−8^
			EUR^d^	170 756; 329 443	0.76	−0.003 (0.005)	1.00 (0.99-1.01)	.53
**Discovery set: studies conducted in East Asian countries**								
rs10240457	7:15431149	A/G	East Asia^e^	12 027; 83 727	0.65	0.028 (0.005)	1.08 (1.05-1.12)	6.48×10^−9^
			EUR^d^	170 756; 329 443	0.50	−0.005 (0.004)	1.00 (0.99-1.00)	.28
**Discovery set: meta-analysis combining ancestrally East Asian and European samples**								
rs7548487	1:177025098	A/G	EAS+EUR^f^	186 527; 508 220	0.90	−0.013 (0.002)	0.96 (0.95-0.98)	1.29×10^−8^
			EAS^c^	15 771; 178 777	0.95	−0.016 (0.007)	0.95 (0.89-1.01)	.02
			EUR^d^	170 756; 329 443	0.88	−0.035 (0.007)	0.97 (0.96-0.97)	1.26×10^−7^
rs547488	18:26481463	C/G	EAS+EUR^f^	186 527; 508 220	0.54	0.008 (0.001)	1.02 (1.01-1.03)	3.25×10^−8^
			EAS^c^	15 771; 178 777	0.78	0.011 (0.004)	1.05 (1.01-1.08)	.003
			EUR^d^	170 756; 329 443	0.45	0.020 (0.004)	1.02 (1.01-1.03)	3.12×10^−6^
rs12160976	22:46438246	A/G	EAS+EUR^f^	186 527; 508 220	0.25	−0.009 (0.002)	0.98 (0.97-0.98)	1.55×10^−8^
			EAS^c^	15 771; 178 777	0.02	−0.026 (0.011)	0.91 (0.81-1.03)	.02
			EUR^d^	170 756; 329 443	0.34	−0.024 (0.005)	0.98 (0.97-0.99)	2.40×10^−7^

Abbreviations: CHR, chromosome; EA, effect allele; EAF, effect allele frequency; EAS, East Asian descent; EUR, European descent; OA, other allele; OR, odds ratio.

^a^ Only the lead variant of each locus is included. The association results for these variants in European ancestry samples from the largest published meta-analysis for depression are also shown.^7^

^b^ Based on an inverse-variance–weighted meta-analysis of the regression coefficients for EAS and EAS+EUR.

^c^ East Asian ancestry GWAS of broad depression outcome.

^d^ Published results from depression GWAS with European ancestry samples.

^e^ Depression GWAS restricted to studies conducted in East Asian countries.

^f^ Meta-analysis between East Asian GWAS^c^ and European ancestry GWAS.^d^

### Association Analyses by Geographic Region and Depression Definition

We further investigated associations by geographic region and by depression definition. We carried out separate meta-analyses in the studies conducted in East Asian countries (12 027 individuals with depression and 83 727 control participants)^20,21,22^ and in studies with ancestrally East Asian participants conducted in the US and the UK (3744 individuals with depression and 95 050 control participants)^23,24,25,26,27,28,29,30,39,40^ (eTable 4 in Supplement 4). A novel locus at 7p21.2 was associated with depression at genome-wide significance in the analysis of the studies conducted in East Asia (Table). The lead SNV, rs10240457 (EAF = 0.646, β for A allele = 0.028, SE = 0.005, *P* = 5.0 × 10^−9^) is intronic to *AGMO* (OMIM 613738). This gene cleaves the O-alkyl bond of ether lipids, which are essential components of brain membranes and function in cell-signaling and other critical biological processes. This variant did not display evidence of association in the samples from studies conducted in the US and UK (β = 0.001, SE = 0.005, *P* = .79) (eFigure 3 in Supplement 2). No other associations were observed at genome-wide significance (eTable 4 in Supplement 4).

We also split the sample to perform outcome-specific analyses (ie, those with clinical diagnosis of depression vs those with self-reported symptoms of depression). No variants were associated at genome-wide significance in the meta-analysis for clinical diagnosis (8223 individuals with depression and 85 370 control participants) nor for symptom-based depression (6124 individuals with depression and 73 095 control participants) (eTable 1 in Supplement 1 and eTable 5 in Supplement 5).

### Meta-analysis of Studies of Participants of East Asian Descent and Studies of Participants of European Descent

We carried out a meta-analysis for the broad depression outcome in cohorts of East Asian descent and the largest GWAS of depression in cohorts of European descent^7^ (Figure 1B; eFigure 4 in Supplement 2). Variants at 43 loci were associated at genome-wide significance. Out of these, 3 loci had not been previously reported, nor did they reach genome-wide significance in either the analysis of European descent cohorts or East Asian descent cohorts alone (Table; eTable 6 in Supplement 6). There was no significant heterogeneity for any of the lead variants at the newly identified loci. The lead variant at 1q25.2, rs7548487 (β for A allele = −0.013, SE = 0.002, *P* = 1.29 × 10^−8^), is located in an intron of *ASTN1* (OMIM 600904). Astrotactin is a neuronal adhesion molecule required for glial-guided migration of young postmitotic neuroblasts in cortical regions of the developing brain.^43^ The C allele of the lead variant at 18q12.1, rs547488, had a β of 0.008 (SE = 0.001) and *P* = 3.3 × 10^−8^. This variant is located downstream of *CDH2* (OMIM 114020), which encodes N-cadherin and has been shown to play a role in the development of the nervous system and be associated with neurodevelopmental disorders.^44^ The third locus is 22q13.31 with lead variant rs12160976 (β for A allele = −0.009, SE = 0.002, *P* = 1.6 × 10^−8^).

### Reproducibility of Depression-Associated Loci

**Figure 2.**
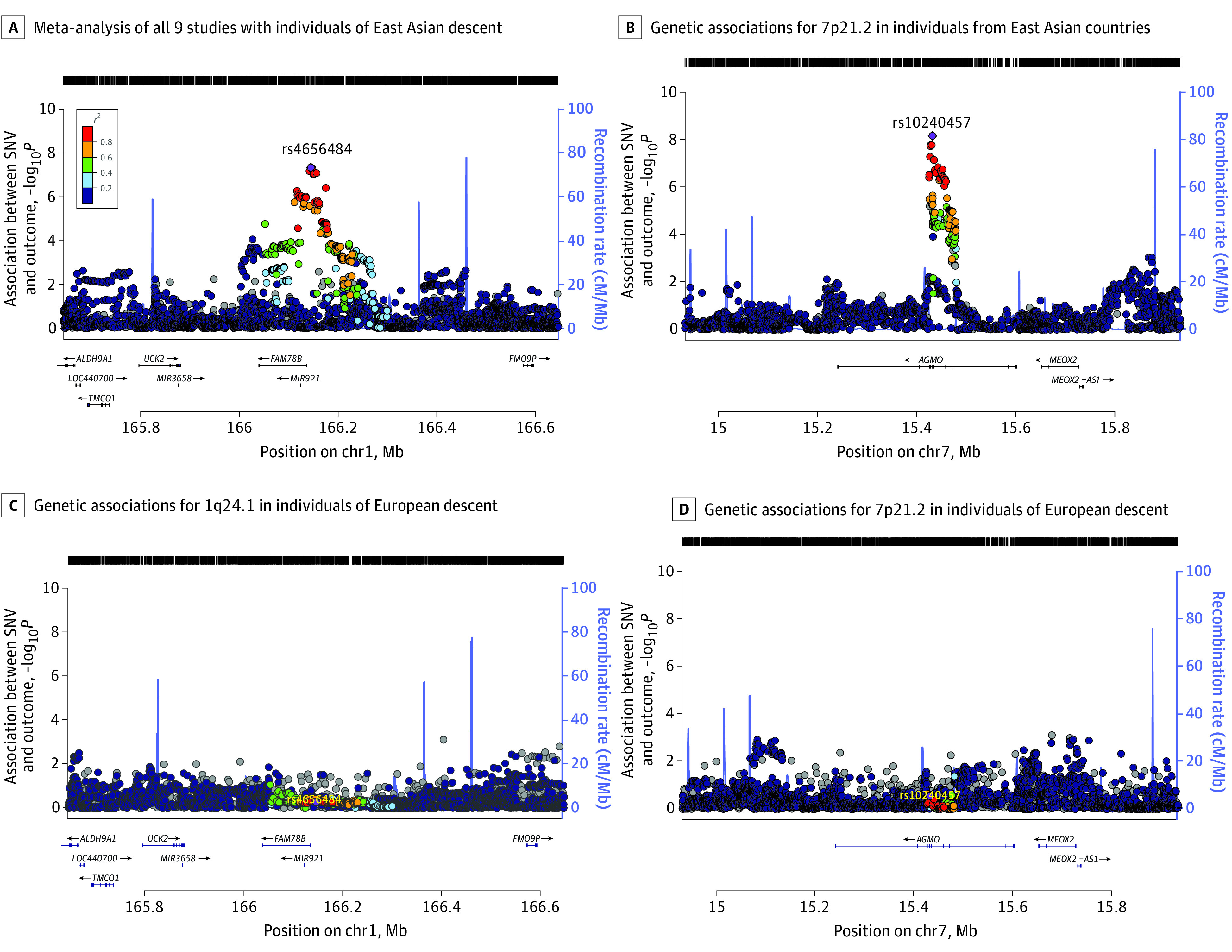
Regional Association Plots of the Depression Associations for 1q24.1 and 7p21.2 The y-axes show the −log_10_*P* values of the association between each SNV and the outcome. The x-axes show the chromosomal position (GRCh37). A Genetic associations for 1q24.1 in the meta-analysis of all 9 studies with ancestrally East Asian samples. B, Genetic associations for 7p21.2 in studies conducted in East Asian countries. C, Genetic associations for 1q24.1 in the largest European depression GWAS.^8^ D, Genetic associations for 7p21.2 based on the largest European depression GWAS.^8^ The purple diamond shows the lead SNV in each region; the color coding depicts the linkage disequilibrium with the lead SNV based on the 1000 Genomes East Asian reference panel.

Although the lead variants of both novel associations from the meta-analyses of individuals of East Asian descent were common in individuals of European descent (EAF = 0.76 and EAF = 0.65 in 1000 Genomes Project phase 3 of individuals of European descent for rs4656484 and rs10240457, respectively), they were not associated with depression in the largest published meta-analysis of depression among individuals of European descent,^7^ and effect sizes similar to those in cohorts of East Asian descent can be ruled out (Table). None of the variants in the credible sets displayed evidence of association at nominal significance levels in the meta-analysis of European ancestry cohorts (Figure 2).

We assessed evidence for reproducibility of previously reported loci for depression. The 2 genome-wide significant loci previously identified in the CONVERGE study^20^ did not show evidence of association in any of the other data sets of cohorts with East Asian ancestry included in this study (eFigure 5 and eTable 7 in Supplement 2). It is worth noting that the effect sizes of these loci in the largest published meta-analysis of depression among individuals of European descent^7^ (eTable 7 in Supplement 2) were also close to 0 for both variants, and the 95% CIs did not overlap with those from CONVERGE (eg, rs12415800 in CONVERGE: β = 0.152; 95% CI = 0.097 to 0.207; European ancestry GWAS: β = −0.004; 95% CI = −0.041 to 0.033).^20^

Of the 102 genetic variants that were independently associated with depression risk in individuals with European ancestry,^7^ 94 lead variants were present in the data for individuals of East Asian ancestry (eTable 8 in Supplement 7). Of these variants, 63 variants (67%) had consistent direction of effect sizes in the European and East Asian ancestry GWASs, more than expected by chance (*P* = .001). Only 11% of these variants were associated with depression at nominal significance in the meta-analysis of cohorts of East Asian descent, although our study was powered to observe 43% under the assumption that the effect sizes are consistent between the cohorts of East Asian descent and the cohorts of European descent (eFigure 6 in Supplement 2). There was no evidence for enrichment of associations at more stringent *P* value thresholds.

For comparison, we also tested how many of the 102 established loci were reproducible in ancestry-matched studies, using several independent European ancestry GWASs with different depression definitions. The expected reproducibility rates varied widely, reflecting the differences in power. The largest data set from 23andMe had a reproducibility rate of 84%, which compared to an expected value of 99% (ratio = 0.86) (eTable 9 in Supplement 2). The lowest reproducibility relative to the expected value was observed for FinnGen, with a ratio of 0.40. However, this was still considerably higher than the ratio of observed vs expected reproducibility for the meta-analysis of cohorts of East Asian ancestry (ratio = 0.25).

### Heritability and Genetic Correlations

The SNV heritability in CONVERGE was 26.2% (SE = 0.03) on the liability scale and 6.4% (SE = 0.02) for CKB based on a prevalence of 6.5%. The clinical diagnosis and symptom-based depression meta-analyses in individuals of East Asian descent had *h*^2^ estimates of 6.8% (SE = 0.02) and 3.8% (SE = 0.04), respectively (eTable 10 in Supplement 2). However, it is likely that depressive symptoms were more common in the population than clinical depression. When we assumed a prevalence estimate of 15%, as in analyses of individuals of European descent, all heritability estimates were significantly increased.

The transancestry genetic correlation between cohorts of East Asian and European descents for clinical depression was *r* = 0.413 (SE = 0.159). We also compared the clinical definition in cohorts of East Asian descent with the symptom-based definition for cohorts of European descent, and the genetic correlation was lower: *r* = 0.223 (SE = 0.181). When using the symptom-based definition for the cohorts of both East Asian and of European descents, we found a correlation of *r* = 0.433 (SE = 0.281). The highest estimate was observed for the comparison of symptom-based depression in individuals of East Asian descent with clinical depression in individuals of European descent: *r* = 0.558 (SE = 0.221). For benchmarking, we also summarized genetic correlations between independent cohorts of East Asian and European descents for other traits and diseases, such as cholesterol, breast cancer, and age at menarche (eTable 11 in Supplement 2). The estimates from large GWASs were consistently higher than the estimates for depression. The genetic correlations for studies with at least 2000 cases ranged from 0.7 to 1. The genetic correlation from the largest study of schizophrenia was *r* = 0.98 (SE = 0.03),^45^ and for bipolar disorder, the correlation was *r* = 0.718 (SE not reported).^46^

**Figure 3.**
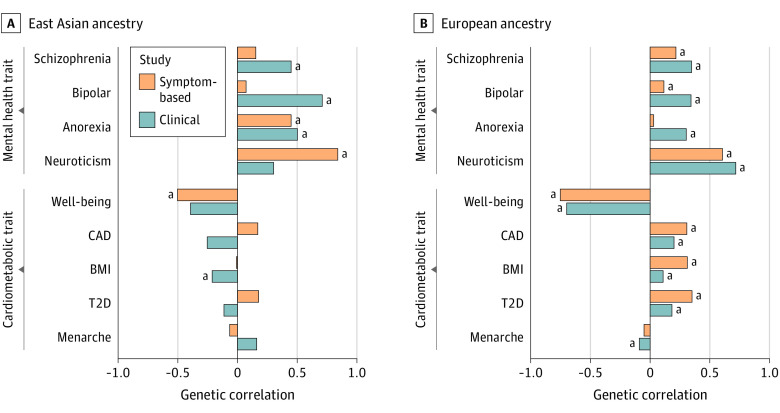
Genetic Correlations for Clinical and Symptom-Based Depression With Cardiometabolic and Mental Health Traits Correlations are shown for samples with East Asian ancestry (A) and European ancestry (B) for the depression studies. For the cardiometabolic and mental health traits, publicly available summary statistics from studies with European ancestry samples were used. Blue bars represent the clinical outcome definition, and orange bars the symptom-based outcome. BMI indicates body mass index; CAD, coronary artery disease; T2D, type 2 diabetes. ^a^Genetic correlations statistically different from zero.

We also assessed the sharing of genetic risk factors between depression in individuals of East Asian descent with other diseases and traits from published summary statistics of studies of individuals of European descent (eTables 12 and 13 in Supplement 2). For clinical depression in individuals of East Asian descent, the highest genetic correlation was observed for bipolar disorder (*r* = 0.710 [SE = 0.153]) (Figure 3).^47^ Clinical depression also had significant positive genetic correlations with other psychiatric disorders, including anorexia nervosa (*r* = 0.502 [SE = 0.158]) and schizophrenia (*r* = 0.449 [SE = 0.109]).^48,49^ For symptom-based depression, the highest correlation was observed for the personality trait of neuroticism (*r* = 0.840 [SE = 0.216]). Symptom-based depression was also negatively correlated with subjective well-being (*r* = −0.502 [SE = 0.195]).^50^

Depression in individuals of European descent has been reported to be genetically correlated with unfavorable cardiometabolic profiles.^6^ However, we observed the opposite for body mass index (BMI) in this study. For clinical depression in individuals of East Asian descent, there was a statistically significant negative genetic correlation with BMI from a GWAS of individuals of European descent (*r* = −0.212 [SE = 0.084]).^51^ The transancestry correlations with type 2 diabetes (T2D) and coronary artery disease were also negative, but not significantly different from 0: *r* = −0.113 (SE = 0.113) and *r* = −0.253 (SE = 0.160, respectively).^52,53^

For a subset of these traits, results for large GWASs of cohorts of East Asian descent were also available. We used these to validate the genetic correlations for depression in individuals of East Asian descent (eFigure 7 and eTable 14 in Supplement 2). For clinically diagnosed depression in individuals of East Asian descent, the estimates were highly consistent for correlations with schizophrenia (*r* = 0.447 [SE = 0.085]), BMI (*r* = −0.147 [SE = 0.061]), and T2D (*r* = −0.143 [SE = 0.072]).^45,54,55^ Correlations between symptom-based depression and the aforementioned traits in individuals of East Asian descent were in the same direction but weaker: schizophrenia (*r* = 0.189 [SE = 0.137]); BMI (*r* = −0.082 [SE = 0.098]); and T2D (*r* = −0.088 [SE = 0.120]).

## Discussion

Herein, we present results of the largest (to our knowledge) GWAS for depression in samples with East Asian ancestry (15 771 individuals with depression and 178 777 control participants). Our results demonstrate the value of combining data from studies with different outcome definitions and study designs, as the increased sample size can empower the discovery of novel associations. Variant rs4656484 at 1q24.1 was associated in studies of individuals of East Asian descent that used different definitions for depression, which suggests that this locus may be linked to the part of the genetic predisposition that is shared between different depression outcomes. Furthermore, by combining GWASs of cohorts of East Asian and European descents, we identified 3 additional novel associations that were not significant in analyses of either the East Asian ancestry cohorts or the European ancestry cohorts alone.

We also observed differences by ancestry, depression outcome definition, and geographic region that highlight the heterogeneity underlying depression. Several depression loci were not transferable between studies of cohorts of East Asian and European ancestry. The newly identified variant rs4656484 was not associated with depression in a previous GWAS of individuals of European descent^7^ (β = 0.003; SE = 0.005; *P* = .53), and an effect size similar to that observed in individuals of East Asian descent can be ruled out. Conversely, only 11% of the established depression loci from studies of participants of European descent were associated with depression at nominal significance in the meta-analysis of individuals of East Asian descent, although the study was powered to observe 43%. The ratio of observed to expected reproducibility was 0.25 for our meta-analysis of individuals of East Asian descent, which was lower than the ratios for several independent ancestry-matched depression GWASs (ratios ranged from 0.40 to 0.86). In line with this, we found moderate transancestry genetic correlations between the depression outcomes in studies of cohorts of East Asian and European descents, ranging from 0.223 to 0.558, consistent with previous findings.^56^ These results are considerably lower than transancestry correlation estimates for other psychiatric traits, such as schizophrenia (*r* = 0.98).^45^ Low transferability could limit downstream applications of depression genetics in transancestry settings, for example in genetic risk prediction.

We also identified a novel depression association at 7p21.2 in studies conducted in East Asian countries. The lead variant was not associated with depression in the US and UK-based data sets, suggesting that nongenetic factors may play an important role for the transferability of loci.^57^ In the context of the growing number of transancestry GWAS meta-analyses, this highlights the importance of considering geographic region as well as genetic ancestry.

Although the genetic risk factors overlap between different depression definitions, their genetic architecture differs, as demonstrated by previous research based on studies of individuals of European descent.^58^ We estimated SNV heritability to be 0.26 in CONVERGE (for severe recurrent depression)^59^ and 0.06 in CKB (for symptom-based depression), which is similar to the previously reported range for different studies of cohorts of European descent of 0.09 to 0.26.^6^ The estimate for CKB supports the hypothesis that lower heritability estimates are linked to less stringent outcome definitions.^58^ However, 0.06 is likely to be an underestimation because the underlying prevalence rate should be higher. In the absence of widely accepted prevalence rates for each of these outcomes in China due to the wide variation in estimates,^60^ we applied the same prevalence estimate for symptom-based and clinical diagnosis definitions of depression.

To account for the differences between clinical and symptom-based depression, we also split our sample and carried out separate association analyses. The genetic correlations with other diseases and traits identified shared and outcome-specific patterns. For clinical depression in individuals of East Asian descent, the highest genetic correlation was observed for bipolar disorder (*r* = 0.710), which was stronger than the respective transancestry genetic correlation with clinical depression in individuals of European descent (*r* = 0.413). For symptom-based depression, on the other hand, the strongest correlation was observed for the personality trait neuroticism (*r* = 0.840). There were also population-specific patterns. The genetic correlations of clinical depression in individuals of East Asian descent with metabolic traits were opposite to that observed for individuals of European descent. European ancestry studies have provided some evidence that BMI is a causal risk factor for major depression.^6^ It is a matter of ongoing research to establish whether this link is due to shared metabolic mechanisms between the 2 phenotypes.^61^ The recruitment strategy in the CONVERGE study, with a high proportion of melancholia subtype and exclusively female participants, may have contributed to the inverse correlation. However, it is unlikely to explain it fully. Symptom-based depression was also inversely correlated with BMI in CKB, but this correlation was not statistically significant. The opposite direction of effect of this risk factor across populations could suggest that the link between depression and weight is social rather than metabolic in nature. This hypothesis is supported by previous work using favorable adiposity genetic variants as an instrument to try to separate the potential biological and social effects of higher adiposity in Europeans.^61^ Genetic variants that are associated with higher adiposity but a more favorable metabolic profile (ie, lower T2D, CAD, and dyslipidemia) were associated with higher odds of depression, suggesting it is not solely the metabolic consequences of higher BMI that drive the association.

In terms of its genetic architecture, major depressive disorder has been shown to be one of the most polygenic outcomes across a wide range of studied phenotypes in cohorts of individuals of European descent^62^ (ie, its potential genetic effects are small and distributed across a very large number of variants in the genome). This is linked to heterogeneity of depression in terms of presentation as well as etiology that results from the complex interplay between genetic and environmental factors.^63,64^ Our results suggest that nongenetic factors, such as cultural differences and other factors, may further add to the heterogeneity of depression and thereby impact on its genetic architecture. First, the spectrum of depression manifestations may overlap but not be identical between cultural contexts of different ancestral groups and geographic regions. Second, many risk factors for depression are determined within a given cultural context and can themselves be heritable, which may modify genetic associations through gene-environment interactions. For example, genetic variants predisposing to higher weight would be associated with depression only in societies where obesity is stigmatized.

### Limitations

This study has some limitations. The data sets we included used different outcome definitions, which can lead to heterogeneity in the meta-analysis. Outcome definitions based on help-seeking behavior may result in a different case group than outcome definitions that fulfill *DSM* criteria for major depressive disorder. More fine-grained conclusions will require greater depth of mental health phenotyping for large samples in future studies. This necessitates global studies in clinical settings as well as general population cohorts with improved mental health phenotyping to address this gap in the future. Some of the studies included in this GWAS meta-analysis used DNA microarrays that were designed for samples from individuals of European descent. These arrays may have lower coverage of the genetic variation present in populations of East Asian descent. General limitations of GWAS apply, as described by Tam et al.^65^ There is a high multiple testing burden. Only a fraction of the heritability is explained by GWAS. Further work is needed to identify the causal variants of the novel associations. Not all genetic determinants of depression can be identified through GWAS. GWAS have largely failed to identify gene-gene interactions. Genetic associations may be influenced by population stratification. The clinical value of GWAS is limited.

## Conclusions

Overall, this study implies caution against generalizing findings about genetic and other risk factors for depression beyond the studied population. It highlights the need for more diverse samples with consistent phenotyping. Increased representation of different populations will benefit locus discovery, fine mapping for potential causal variants, and polygenic risk score profiling and could help address health disparities.^57,66,67,68,69^

## Notes

Supplement 1. eTable 1. Descriptive characteristics of the datasets included in this study

Supplement 2. eAppendix 1. Additional Information eFigure 1. Quantile-quantile Plot Illustrating the GWAS Meta-Analysis for Depression in 15,771 Individuals With Depression and 178,777 Individuals with East Asian Ancestry eFigure 2. Forest Plot for rs4656484 Which Was Genome-Wide Significant in the Depression East Asian Meta-Analysis Based on all Studies eFigure 3. Forest Plots of rs10240457 Which Was Genome-Wide Significant in the Depression Meta-Analysis of Studies Based in East Asian Countries eFigure 4. Quantile-Quantile Plot Illustrating the Meta-Analysis of East Asian Results for Depression With the Largest GWAS in Europeans (Howard et al., 2019) eFigure 5. Forest Plots for the Two Previously Reported Depression Loci Based on the Chinese CONVERGE Study eFigure 6. Effect Estimates for Depression of Previously Reported Depression Loci in the Discovery European Study vs in the East Asian Meta-Analysis eFigure 7. Genetic Correlations Between the Clinical and Symptom-Based Depression Phenotypes in East Asians and Other Traits in Europeans eTable 1. Descriptive Characteristics of the Datasets Included in This Study provided in the separate excel file eTable 2. Details of the Genotyping for Each Dataset Included in the Discovery East Asian Meta-Analysis for Depression eTable 3. Variants Significantly Associated With Depression (P<10-5) in the East Asian Discovery Meta-Analysis and Their Results in the Largest Depression GWAS in Europeans (Howard et al., 2019) provided in the separate excel file eTable 4. Variants Associated at P<10-5 in the Depression GWAS Restricted to East Asia Based Studies vs Their Results in the USA/UK-Based Studies provided in the separate excel file eTable 5. Loci That Reached the Suggestive Significance Threshold (P<10-5) in Either of the Outcome-Specific Analyses in East Asian Ancestry Samples provided in the separate excel file eTable 6. Variants Associated With Depression (P<5x10-8) in the Meta-Analysis of the Broad Depression Outcome in East Asian Ancestry Samples and the Largest Depression GWAS in European Ancestry Samples (Howard et al., 2019) provided in the separate excel file eTable 7. The Two Genome-Wide Significant Depression Loci in CONVERGE and the Relevant Results in the Other East Asian Ancestry Datasets of the Current Study and the Two Largest European Studies eTable 8. The Association of Previously Reported Variants for Depression With P<5x10-8 in European Ancestry Samples (Howard et al., 2019) in the East Asian Discovery Meta-Analysis provided in the separate excel file eTable 9. Reproducibility of Established Depression Loci From Howard et al.,2019 in Independent Samples of European Ancestry and in the East Asian Depression GWAS eTable 10. SNP-Heritabilities for the Different Depression-Definition Outcomes Considered in This Study eTable 11. Transancestry Genetic Correlation Estimates for Diverse Phenotypes Between Samples With East Asian and European Ancestry eTable 12. Transancestry Genetic Correlations of Clinical and Symptom-Based Depression in East Asian Datasets With Other Traits, Based on European Studies eTable 13. Genetic Correlations of Clinical and Symptom-Based Depression in European Datasets With Other Traits eTable 14. Genetic Correlations of Clinical and Symptom-Based Depression in East Asian Datasets With Other Traits, Based on East Asian Studies eReferences

Supplement 3. eTable 3. Variants significantly associated with depression (P<10-5) in the East-Asian discovery meta-analysis and their results in the largest depression GWAS in Europeans

Supplement 4. eTable 4. Variants associated at P<10-5 in the depression GWAS restricted to East-Asia based studies vs their results in the USA/UK-based studies

Supplement 5. eTable 5. Loci that reached the suggestive significance threshold (P<10-5) in either of the outcome-specific analyses in East-Asian ancestry samples

Supplement 6. eTable 6. Variants associated with depression (P<5x10-8) in the meta-analysis of the broad depression outcome in East-Asian ancestry samples and the largest depression

Supplement 7. eTable 8. The association of previously reported variants for depression with P<5x10-8 in European ancestry samples (Howard et al., 2019) in the East-Asian discovery meta-analysis

Supplement 8. Nonauthor Collaborators
