# Supplementary material for: The Genetic Architecture of Depression in Individuals of East Asian Ancestry: A Genome-Wide Association Study
Source: JAMA Psychiatry. 2021 Sep 29;78(11):1–12. doi: 10.1001/jamapsychiatry.2021.2099 (PMC8482304; doi:10.1001/jamapsychiatry.2021.2099)
Supplement: Supplement 2. — eAppendix 1. Additional Information eFigure 1. Quantile-quantile Plot Illustrating the GWAS Meta-Analysis for Depression in 15,771 Individuals With Depression and 178,777 Individuals with East Asian Ancestry eFigure 2. Forest Plot for rs4656484 Which Was Genome-Wide Significant in the Depression East Asian Meta-Analysis Based on all Studies eFigure 3. Forest Plots of rs10240457 Which Was Genome-Wide Significant in the Depression Meta-Analysis of Studies Based in East Asian Countries eFigure 4. Quantile-Quantile Plot Illustrating the Meta-Analysis of East Asian Results for Depression With the Largest GWAS in Europeans (Howard et al., 2019) eFigure 5. Forest Plots for the Two Previously Reported Depression Loci Based on the Chinese CONVERGE Study eFigure 6. Effect Estimates for Depression of Previously Reported Depression Loci in the Discovery European Study vs in the East Asian Meta-Analysis eFigure 7. Genetic Correlations Between the Clinical and Symptom-Based Depression Phenotypes in East Asians and Other Traits in Europeans eTable 1. Descriptive Characteristics of the Datasets Included in This Study provided in the separate excel file eTable 2. Details of the Genotyping for Each Dataset Included in the Discovery East Asian Meta-Analysis for Depression eTable 3. Variants Significantly Associated With Depression (P<10-5) in the East Asian Discovery Meta-Analysis and Their Results in the Largest Depression GWAS in Europeans (Howard et al., 2019) provided in the separate excel file eTable 4. Variants Associated at P<10-5 in the Depression GWAS Restricted to East Asia Based Studies vs Their Results in the USA/UK-Based Studies provided in the separate excel file eTable 5. Loci That Reached the Suggestive Significance Threshold (P<10-5) in Either of the Outcome-Specific Analyses in East Asian Ancestry Samples provided in the separate excel file eTable 6. Variants Associated With Depression (P<5x10^-8^) in the Meta-Analysis of the Broad Depression Outcome in East Asian Ance [file jamapsychiatry-e212099-s002.pdf]

Supplementary Online Content

Giannakopoulou O, Lin K, Meng X, et al; for the 23andMe Research Team, China Kadoorie Biobank Collaborative Group, and Major Depressive Disorder Working Group of the Psychiatric Genomics Consortium. The genetic architecture of depression in individuals of East Asian ancestry. Published online September 29, 2021. *JAMA Psychiatr*. doi:10.1001/jamapsychiatry.2021.2099

eAppendix 1. Additional Information

eFigure 1. Quantile-quantile Plot Illustrating the GWAS Meta-Analysis for Depression in 15,771 Individuals With Depression and 178,777 Individuals with East Asian Ancestry

eFigure 2. Forest Plot for rs4656484 Which Was Genome-Wide Significant in the Depression East Asian Meta-Analysis Based on all Studies

eFigure 3. Forest Plots of rs10240457 Which Was Genome-Wide Significant in the Depression Meta-Analysis of Studies Based in East Asian Countries

eFigure 4. Quantile-Quantile Plot Illustrating the Meta-Analysis of East Asian Results for Depression With the Largest GWAS in Europeans (Howard et al., 2019)

eFigure 5. Forest Plots for the Two Previously Reported Depression Loci Based on the Chinese CONVERGE Study

eFigure 6. Effect Estimates for Depression of Previously Reported Depression Loci in the Discovery European Study vs in the East Asian Meta-Analysis

eFigure 7. Genetic Correlations Between the Clinical and Symptom-Based Depression Phenotypes in East Asians and Other Traits in Europeans

eTable 1. Descriptive Characteristics of the Datasets Included in This Study *provided in the separate excel file*

eTable 2. Details of the Genotyping for Each Dataset Included in the Discovery East Asian Meta-Analysis for Depression

eTable 3. Variants Significantly Associated With Depression ($P<10^{-5}$) in the East Asian Discovery Meta-Analysis and Their Results in the Largest Depression GWAS in Europeans (Howard et al., 2019) *provided in the separate excel file*

eTable 4. Variants Associated at $P<10^{-5}$ in the Depression GWAS Restricted to East Asia Based Studies vs Their Results in the USA/UK-Based Studies *provided in the separate excel file*

eTable 5. Loci That Reached the Suggestive Significance Threshold ($P<10^{-5}$) in Either of the Outcome-Specific Analyses in East Asian Ancestry Samples *provided in the separate excel file*

© 2021 Giannakopoulou O et al. *JAMA Psychiatry*.

eTable 6. Variants Associated With Depression ($P<5x10^{-8}$) in the Meta-Analysis of the Broad Depression Outcome in East Asian Ancestry Samples and the Largest Depression GWAS in European Ancestry Samples (Howard et al., 2019) *provided in the separate excel file*

eTable 7. The Two Genome-Wide Significant Depression Loci in CONVERGE and the Relevant Results in the Other East Asian Ancestry Datasets of the Current Study and the Two Largest European Studies

eTable 8. The Association of Previously Reported Variants for Depression With $P<5x10^{-8}$ in European Ancestry Samples (Howard et al., 2019) in the East Asian Discovery Meta-Analysis *provided in the separate excel file*

eTable 9. Reproducibility of Established Depression Loci From Howard et al.,2019 in Independent Samples of European Ancestry and in the East Asian Depression GWAS

eTable 10. SNP-Heritabilities for the Different Depression-Definition Outcomes Considered in This Study

eTable 11. Transancestry Genetic Correlation Estimates for Diverse Phenotypes Between Samples With East Asian and European Ancestry

eTable 12. Transancestry Genetic Correlations of Clinical and Symptom-Based Depression in East Asian Datasets With Other Traits, Based on European Studies

eTable 13. Genetic Correlations of Clinical and Symptom-Based Depression in European Datasets With Other Traits

eTable 14. Genetic Correlations of Clinical and Symptom-Based Depression in East Asian Datasets With Other Traits, Based on East Asian Studies

eReferences

This supplementary material has been provided by the authors to give readers additional information about their work.

© 2021 Giannakopoulou O et al. *JAMA Psychiatry*.

eAppendix 1. Additional Information

Supplementary Methods

Raw genotype data were available for China Kadoorie Biobank (CKB), UK Biobank (UKB), Intern Health Study (IHS), Women's Health Initiative (WHI) and Army Study To Assess Risk and Resilience in Service members (Army-STARRS) study. Summary statistics were available for China Oxford and VCU Experimental Research on Genetic Epidemiology (CONVERGE) study, Bio*Me*, Taiwan-Major Depressive Disorder (MDD) study and 23andMe cohort. We did not assess whether there is sample overlap between studies because given their different design and area of recruitment sample overlap is extremely unlikely.

This investigation was based on EAS participants which was defined by the investigators based on genetic information. For each study a principal component analysis was carried out based on the genetic similarity of pairs of individuals. Individuals that clustered around a reference group with confirmed East Asian ancestry were included in this analysis.

<u>Studies description</u>

A. China Kadoorie Biobank (CKB)

The CKB is a large population dataset of more than 510,000 individuals from 10 geographically defined regions of China, with extensive clinical, lifestyle, dietary, medical history and genetic data[1]. All the participants were interviewed at the baseline by trained staff, while periodic re-surveys have been conducted in ~25,000 surviving participants. Health outcomes of the participants provided through linkages with established registries and health insurance databases are also available.

A total of 17,723 participants reported that had experienced at least one Composite International Diagnostic Interview (CIDI) -A trigger symptom (i.e., feeling sad/depressed, loss of appetite, loss of interest or feeling worthless) for two or more weeks during the past year and were categorized as having "symptom-based" depression in our analyses. These participants were further assessed for major depression (MD) using the Chinese version of the CIDI-short form by trained clinicians at study clinics. Participants were defined as having past year MD if they had felt sad, blue, or depressed for ≥2 weeks during the past 12 months, accompanied by at least 3 of 7 additional symptoms, including weight/appetite change, sleep problems, loss of interest and pleasure, loss of energy or fatigue, concentration problems, feelings of guilt or worthlessness, and thoughts of suicide. In our analysis, 4,500 participants who fulfilled the past year MD CIDI-criteria, had at least one relevant medical record (ICD10 F32, F33, F34.1, F38.1 codes) during the follow-up period or reported at resurvey 2 that have been ever diagnosed by a doctor with depression were classified as having "lifetime diagnosis of MD". Participants that had never been diagnosed with MDD (either diagnosed by CIDI-A questionnaire, self-reported depression or had a medical diagnosis of depression (F32, F33, F34.1, F38.1)) or with neurasthenia and did not report any MDD symptoms constituted the control group (~70,000) in all our analyses. Further exclusions from both cases and controls groups included participants with a medical diagnosis for dementia, psychosis, bipolar disorder, mental retardation and pervasive developmental disorders.

A total of 102,783 participants have been genotyped using 2 custom-designed Affymetrix Axiom arrays including up to 803,000 variants, optimised for genome-wide coverage in Chinese populations. Stringent quality control (QC) included SNP call rate > 0.98, plate effect $P > 10^{-6}$, batch effect $P > 10^{-6}$, Hardy-Weinberg Equilibrium (HWE) deviations $P > 10^{-6}$ (combined 10df $\chi 2$ test from 10 regions), biallelic, Minor Allele Frequency (MAF) difference from 1000 Genomes East-Asian frequencies < 0.2, sample call rate > 0.95, heterozygosity < mean + 3 standard deviation (SD), no sex chromosomes aneuploidy, genetically-determined sex concordant with database, resulting in genotypes for 532,415 variants present on both array versions. Genotypes were imputed to the 1,000 Genomes Phase 3 reference (EAS MAF > 0) using SHAPEIT version 3 and IMPUTE version 4.

A total of 5,376 symptom-based depression cases (1,305 participants with lifetime diagnosis) and 69,998 controls have been genotyped. A linear mixed model (SAIGE) was implemented for the association with depression, adjusting

© 2021 Giannakopoulou O et al. *JAMA Psychiatry.*

for age, sex, principal components (PCs) and recruitment region. After filtering variants with effective sample size (Neff) < 50^2 and poorly imputed variants (info<0.7), 10,834,708 variants were included in the downstream analyses.

B. China, Oxford and Virginia Commonwealth University Experimental Research on Genetic Epidemiology cohort (CONVERGE)

The CONVERGE cohort of Han Chinese women has been previously described[3]. Briefly, ~5,000 cases of recurrent MDD (≥2 episodes), established with the CIDI, which used DSM-IV criteria, were analysed against an equal number of controls. Cases with medical history of bipolar disorder, psychosis, mental retardation and/or drug or alcohol abuse before their first depressive episode were excluded from the study.

CONVERGE samples underwent whole-genome sequencing, as previously described[3]. In brief, after genotyping calling, two rounds of imputation were performed: first without a reference panel and then using the 1000Genomes Phase 1 Asian haplotypes. Variants with a) a P-value for violation HWE < 10^{-6}, b) information score < 0.9 and c) MAF in CONVERGE < 0.5% were excluded from the GWAS, resulting in a final set of 5,987,610 SNPs. The GWAS was conducted with a mixed-linear model including a genetic relationship matrix (FastLMM version 2.06.20130802) as random effect and PCs from eigen-decomposition of this matrix as fixed effects. We further filtered the publicly available GWAS summary statistics by removing variants with Neff less than 50.

C. 23andMe cohort

The GWAS dataset of personal genetic company 23andMe, Inc. (Sunnyvale, CA) that included in this meta-analysis, encompassed 2,729 depression cases and 90,310 controls of East Asian ancestry. All participants provided informed consent and answered surveys online according to 23andMe's human subject protocol, which was received and approved by Ethical & Independent Review Services, an AAHRPP-accredited institutional review board. As part the medical history survey, participants were asked if they have ever received a clinical diagnosis or treatment for depression (binary variable).

DNA extraction and genotyping were performed on saliva samples by National Genetics Institute (NGI), a CLIA licensed clinical laboratory and a subsidiary of Laboratory Corporation of America. Samples were genotyped on one of five genotyping platforms. The v1 and v2 platforms were variants of the Illumina HumanHap550+ BeadChip, including about 25,000 custom SNPs selected by 23andMe, with a total of about 560,000 SNPs. The v3 platform was based on the Illumina OmniExpress+ BeadChip, with custom content to improve the overlap with our v2 array, with a total of about 950,000 SNPs. The v4 platform was a fully customized array, including a lower redundancy subset of v2 and v3 SNPs with additional coverage of lower-frequency coding variation, and about 570,000 SNPs. The v5 platform (68.4% of the samples in the East-Asian dataset), is an Illumina Infinium Global Screening Array (~640,000 SNPs) supplemented with ~50,000 SNPs of custom content. This array was specifically designed to better capture global genetic diversity and to help standardize the platform for genetic research.

Imputation was performed with Minimac3 using a reference panel combining the May 2015 release of the 1000 Genomes Phase 3 haplotypes with the UK10 imputation reference panel. The association testing was performed by logistic regression assuming additive allelic effects, adjusting for age, sex, the top five principal components to account for residual population structure and indicators for genotype platforms to account for genotype batch effects. The association analysis and the downstream quality control was conducted separately for the genotyped and the imputed SNPs.

Genotyped GWAS results were filtered for: SNPs that were only genotyped on "v1" and/or "v2" platforms due to small sample size, SNPs on chrM or chrY, SNPs that failed a test for parent-offspring transmission, SNPs with fitted $\beta<0.6$ and $P<10^{-20}$ for a test of $\beta<1$, SNPs with a Hardy-Weinberg $P<10^{-20}$, or a call rate of <90%, SNPs with genotype date effects (determined as $P<10^{-50}$ by ANOVA of SNP genotypes against a factor dividing genotyping date into 20 roughly equal-sized buckets), SNPs with large sex effect (ANOVA of SNP genotypes, r2>0.1), SNPs with probes matching multiple genomic positions in the reference genome and variants with minor allele counts in the controls less than 50.

© 2021 Giannakopoulou O et al. *JAMA Psychiatry*.

For imputed GWAS results, SNPs with poor imputation quality (rsq<0.7), Neff less than 50 and SNPs that had strong evidence of a platform batch effect were excluded from the downstream analysis. The batch effect test is an F test from an ANOVA of the SNP dosages against a factor representing v4 or v5 platform ($P<10^{-50}$).

Across all results, further filtering was performed on SNPs that have an available sample size of less than 20% of the total GWAS sample size, logistic regression results that did not converge due to complete separation, identified by abs(effect)>10 or stderr>10 on the log odds scale.

D. Taiwan-Major Depressive Disorder (MDD) Study

MDD patients were included from a family study of mood disorders in Taiwan. Patients aged between 18 to 70 years, who met diagnostic criteria of MDD using the Diagnostic and Statistical Manual of Mental Disorders, fourth edition (DSM-IV) were consecutively referred by psychiatrists in clinical settings. Exclusion criteria include patients diagnosed with schizophrenia, schizoaffective or substance-induced mood disorders. The community-recruited Taiwan Biobank Dataset was used as the control group. The control group was further filtered by excluding subjects who have self-reported bipolar disorder (BPD), postpartum depression, alcoholism or drug addiction, schizophrenia, Parkinson's disease or dementia. Moreover, subjects in Taiwan Biobank Data who had self-reported diagnosis of MDD were classified into case (MDD) group. Sample collection procedures and detailed information about Taiwan Biobank were described elsewhere[4]. Both MDD and control subjects were Han Chinese.

Genotyping for Taiwan MDD cases was obtained using Affymetrix CHB Array with 642,832 genetic variants, Affymetrix TWB1.0 Array with 642,545 variants, Illumina Human Omni Express Exiome Beadchips with 949,974 variants and Affymetrix TWB2.0 Array with 689,688 variants. Genotyping for Taiwan Biobank controls was obtained using Affymetrix TWB1.0 Array with 646,735 variants and Affymetrix TWB2.0 Array with 686,439 variants. Owing to Affymetrix TWB2.0 is a very unique array specifically designed for Taiwanese, which is very different from other platforms, imputation was performed separately. One set of imputation was done for Affymetrix TWB2.0 array only, the other set of the imputation was done with combining all other arrays, including Affymetrix CHB, TWB1.0, and Illumina arrays using common SNPs across platforms. The overlapping variants of Affymetrix TWB2.0 array and all other platforms were around 28,000. Imputation was conducted by Michigan Imputation Server using 1000G phase 3 v5 as a reference panel, Eagle v2.3 for phasing, and EAS population for QC. Samples that did not meet the 95% threshold of call rate, kinship-pairs and outliers in population stratification were removed. Genetic variants with call rate <95%, MAF <0.01, p-value of HWE $<10^{-6}$ were also excluded. The GWAS was performed using PLINK 1.9 and adjusted for 5 ancestry principal components. The GWA analysis was conducted separately by platforms with (1) Affymetrix TWB2.0 and (2) all other platforms combined together. In the latter, variants significantly associated (P<0.005) with genotyping platforms were excluded from downstream analysis. We also used a stricter imputation threshold for filtering (info<0.9 instead of 0.7).

E. Women's Health Initiative study (WHI)

The WHI study is a long-term national health study in U.S conducted in postmenopausal women, enrolled either in a clinical trial or an observational study[5]. We analysed data from 3,492 women with Asian ancestry who were genotyped as part of the WHI – Population Architecture using Genomics and Epidemiology (PAGE) sub-study. These participants had agreed their data to be included in the database of Genotypes and Phenotypes (dbGaP). The genotype and phenotype data were assessed vid dbGaP study accession phs000200.v12.p3. Depressive symptoms in the past week were assessed in the baseline visit with a 6-item Center for Epidemiological Studies Depression Scale (CES-D) form. Based on Smoller et al., definitions[6], participants with a score of 5 or more were considered as depression cases, while participants not classified as currently depressed (6-item CES-D), without medical history of depression (2-item Diagnostic Interview Schedule) and not on antidepressant therapy constituted the control group.

The dataset of Asian participants of WHI included in our analyses, have been genotyped with CardioMetaboChip, as part of the NHGRI's PAGE project. Samples and variants with a call rate lower than 95%, typed variants with different missingness rates between case and control group > 0.2 and variants with MAF < 0.05 were excluded from downstream analysis. A logistic regression analysis was performed (PLINK2), adjusting for age, sex, 20 PCs and study subgroup.

© 2021 Giannakopoulou O et al. *JAMA Psychiatry*.

F. Intern Health Study (IHS)

We also considered participants from IHS, a multi-institutional longitudinal cohort study of medical interns in U.S. The study design has been previously described[7]. Depressive symptoms were measured through the PHQ-9 questionnaire, a self-report component of the primary care evaluation of mental disorders inventory. Subjects were asked to complete the questionnaire assessing PHQ-9 depressive symptoms in the baseline survey, as well as at months 3, 6, 9 and 12 of their internship year. Participants with a PHQ-9 score of 10 or greater[8] during their internship were considered as depression cases in this study. A total of 294 depression cases and 544 controls were considered in this association study.

IHS samples were genotyped on Illumina Infinium CoreExome v1.0 or v1.1 array. Quality control steps and imputation were performed using the Ricopili Rapid Imputation Consortium Pipeline[9]. Study samples were assigned into distinct ancestry groups based on PCs derived from the study samples combined with 1000Genomes reference panel. In brief, samples with call rate < 98% or samples with a gender mismatch between genotype and reported data were excluded. For duplicated samples and up to third-degree relatives, the sample with higher call rate was selected. Variants with call rate < 98%, missing difference > 0.20 were also excluded prior imputation. Genotypes were imputed to the Haplotype Reference Consortium (HRC) reference panel using EAGLE and IMPUTE2 for the phasing and the imputation respectively. A logistic regression analysis was performed (PLINK2) in genotype dosages, adjusting for age, sex, 20 first PCs. Variants with MAF < 0.05 and imputation info score < 0.7 were excluded from downstream analysis, resulting in a dataset of 4,626,568 variants.

G. UK Biobank (UKB)

UKB is a well-characterized cohort of more than 500,000 individuals recruited at UK between 2006-2010 with linked health and genetic data[10]. A subset of participants has also completed the mental-health questionnaire. We used a combination of hospital diagnoses (ICD10 codes) and lifetime CIDI (A. prolonged feelings of depression OR prolonged loss of interest in normal activities AND B. affected more than half of the day during worst episode of depression AND C. the frequency of depressed days during worst episode was at almost every day/every day AND D. these problems interfered with your life/activities (study/employment, childcare and housework, leisure pursuits) somewhat/a lot) to define our cases. Gender mismatches, missingness/heterozygosity outliers, participants with excessive genetic relatedness, no quality control metrics, individuals that have withdrawn their consent and up to 2nd degree relatives (PC-Relate) were excluded before the analysis.

UKB genotyping was conducted by Affymetrix using two similar arrays; Applied Biosystems™ UK BiLEVE Axiom™ Array, consisting of 807,411 genetic variants and a bespoke UK Biobank Axiom™ array, including 825,927 genetic variants. All genetic data was quality controlled by UKBB bioinformatics team, both at sample and marker level, resulting in a dataset of 488,377 samples and 805,426 variants from both arrays. The genetic data was subsequently imputed by UKB to over 90 million SNPs, indels and large structural variants, using haplotypes of both British, European and diverse-ancestry populations. For this study, we used data imputed with both the HRC and the merged UK10K and 1000Genomes Phase 3 reference panels[10]. To assign individuals in ancestry groups based on their genetic information, we implemented the PC-AiR method to perform a PC analysis for the detection of population structure[11]. A logistic regression analysis was performed in imputed genetic dataset (PLINK2), adjusting for age, sex, genotyping array and PCs that were calculated based on the subset of genetically defined EAS participants. Downstream analysis was restricted in the subset of common (MAF > 0.05) and well-imputed (> 0.7) variants. The analysis conducted under UK Biobank application 51119.

H. Army Study To Assess Risk and Resilience in Service members (Army STARRS) study

Data from the Army-STARRS, a study conducted in army members in USA, were also assessed in the current analysis. Army STARRS includes the New Soldier Study (NSS) and the Pre/Pst Deployment Study (PPDS). Detailed information about the design of the study have been published previously[12]. Depression outcomes were measured with the CIDI screening scales and evaluated for concordance with DSM-IV diagnoses within the Army STARRS clinical reappraisal study[13].

© 2021 Giannakopoulou O et al. *JAMA Psychiatry.*

The genotyping and imputation of Army-STARRS, New Soldier Study (NSS) samples has been described previously[14]. In brief, samples were genotyped using the Illumina OmniExpress and Exome array and were imputed on a reference multi-ancestry panel from the 1000G Genomes Project (phase1). Samples and genetic variants with a call rate less than 95% and 98% respectively were filtered out. A logistic regression analysis was performed in common and well-imputed variants (PLINK2), adjusting for age, sex and the 20 first PCs.

I. Bio*Me*

Bio*Me* is an electronic medical record-linked biobank of more than 50,00 participants from the Mount Sinai Health System[15]. Bio*Me* cases were individuals with a medical depression diagnosis (ICD9 296.2, 296.3, 296.82, 298.0, 300.4, 301.12, 311; ICD10 F32, F33, F34.1, F38.1). Participants diagnosed with dementia, bipolar or manic disorder, developmental disorders, intellectual disability, psychotic disorder, personality disorder were excluded from this study.

Bio*Me* samples were genotyped with the Infinium Global Screening Array (GSA) BeadChip. Individuals with population-specific heterozygosity rate that surpassed +/- 6 standard deviations of the population-specific mean, along with individuals with a call rate of <95%, individuals with discordant reported and genetic sex and with phenotypically intermediate sex were not considered in the analysis. In cases of duplicates, the sample of each pair with the lower missingness rate in the exomic data was preferentially excluded. Genetic variants exclusions included a call rate <95% and HWE $p < 10^{-5}$. The resulting dataset was imputed to the 100Genomes Phase 3 reference panel. The GWAS was performed with a binary mixed model (SAIGE). The first 20 PCs were calculated using PLINK (v1.9) and a genomic relationship matrix (GRM) was calculated using the KING (v1.4) software (-ibs). The PCA and GRM calculations were restricted to common (MAF>0.01), autosomal sites. Additionally, variants with MAF<0.05 and info<0.07 were excluded before the meta-analysis.

Data availability statement

Summary statistics for the combined EAS meta-analysis excluding the 23andMe study are available through the PGC website (http://www.med.unc.edu/pgc/downloads). The genome-wide summary statistics for CONVERGE and the European meta-analysis are also available on the PGC website. Uploading and sharing of individual genetic data from CKB are subject to restrictions according to the Interim Measures for the Administration of Human Genetic Resources administered by the Human Genetic Resources Administration of China (HGRAC). Summary data including allele frequencies and GWAS summary statistics are available by application and restricted to research-related purposes. Other individual-level CKB data are available through www.ckbiobank.org, subject to completion of a Material Transfer Agreement, either through Open Access or on application. CKB data access is subject to oversight by an independent Data Access Committee. Analyses using CKB data were conducted under research approval 2018-0018. Data from 23andMe, Inc were made available under a data use agreement that protects participant privacy. Please visit https://research.23andme.com/collaborate/#dataset-access for more information and to apply to access the data. The raw genetic and phenotypic UK Biobank data used in this study, which were used under license (application number 51119), are available from: http://www.ukbiobank.ac.uk/. The genotype and phenotype data for the WHI study can be requested via dbGaP study accession phs000200.v12.p3.

Genotyping

The genotyping of each study has been previously described [3, 4, 10, 14, 16]. To optimise genome-wide coverage in EAS populations, genotyping was carried out using two custom-designed Affymetrix Axiom arrays in CKB and the Affymetrix TWB2.0 array for a subset of the Taiwan-Major Depressive Disorder study samples [1, 4]. CONVERGE used whole-genome sequencing with a mean depth of 1.7[3]. More detail for all studies is provided in the studies description above.

© 2021 Giannakopoulou O et al. *JAMA Psychiatry.*

Quality control

Quality control and association analyses were carried out separately for each study as described in the studies description and Supplementary Table 2. Genotypes were imputed to 1000 Genomes Project reference panel, except IHS where the Haplotype Reference Consortium (HRC) was used, 23andMe and UKB where the 1000 Genomes data were combined with the UK10K and HRC imputation reference panel, respectively. In the meta-analysis, we included only well-imputed variants (imputation accuracy > 0.7) with effective sample size (N_{eff}) equal or higher than 50^2 in the larger datasets (CONVERGE, CKB, 23andMe), and with minor allele frequency (MAF)>=0.05 in the other studies. For the Taiwan-MDD study an imputation accuracy threshold of 0.9 was used.

Meta-analysis

We performed a Z-score weighted meta-analysis using METAL[33] for 13,163,200 genetic variants (Supplementary Figure 1). For all meta-analyses, results were restricted to variants present in at least two studies. We also performed a Z-score weighted meta-analysis combining results from our EAS analysis and the publicly available summary statistics from the largest published GWAS in EUR samples[17]. Variants associated at genome-wide significance in this trans-ancestry meta-analysis were considered novel if they were located outside ±250kb either side of the lead variants from the published GWAS of depression in EUR and if the Linkage Disequilibrium (LD) with the lead variant was <0.01 [17]. We calculated betas for the meta-analyses using the formula from Zhu et al.[18]. Odds ratios were based on an inverse-variance weighted meta-analysis of the study betas, where for CONVERGE we used results from a logistic regression in Plink instead of FastLMM.

Functional annotation and gene-based association analysis

We functionally annotated the lead variants and their proxies ($r^2 \geq 0.8$). Gene-based association analysis was performed using MAGMA (v1.08), implemented in FUMA, with default settings[19, 20]. SNPs were mapped to 19,575 protein coding genes from Ensembl build 85. Significance for the gene-based analysis was defined as the Bonferroni corrected threshold ($P=2.6x10^{-6}$).

We functionally annotated the lead SNPs in the genomic regions associated with increased risk for depression using HaploReg v4[21] and Open Targets Genetics Platfrom[22]. Candidate genes for each locus associated with depression were selected based on their proximity to the lead variant and/or the evidence of eQTL associations for a gene in that region. Open Targets Genetics interrogates various data sources to link genetic variation to genetic expression. The GeneCards database was used to obtain summary information of the identified genes, while NCBI's PubMed database was used to interrogate literature related to gene function and association with other human traits/diseases. We queried the identified variants and their proxies in PhenoScanner[23] and the NHGRI-EBI GWAS catalogue[24] to investigate trait pleiotropy.

Reproducibility of established depression loci

We assessed whether the associations of 102 established depression loci from the largest published EUR GWAS [17] were reproducible in samples with EAS ancestry. Since the lead SNP might not be the causal variant nor correlated with it in other ancestry groups due to LD differences, we also formed credible sets that are likely to include the causal variant. These were based on all variants in LD with the lead variant of a locus ($r^2>0.6$) based on an ancestry matched reference (1000 Genomes Project v3 EUR samples). We assessed whether any variant in the credible set displayed evidence of association in the target study. As these credible sets contained multiple SNPs, we used a p-value threshold of P<0.01 to indicate reproducibility. While this p-value threshold might not provide conclusive evidence of reproducibility for individual loci, we used it to test reproducibility rates across sets of loci.

© 2021 Giannakopoulou O et al. *JAMA Psychiatry*.

We estimated the number of associations out of the 102 established loci that were expected to replicate. We accounted for the sample size of our study and the allele frequency in EAS populations. First, we calculated the power[25] to observe an association in the EAS meta-analysis for each of the 102 loci at alpha error of 0.05 using the effect estimate from the EUR discovery study[8], the allele frequency for EAS samples from 1000 Genomes and the sample size available in the EAS meta-analysis. By summing up the probabilities across the 102 loci, we derived the absolute number of associations out of the 102 we are powered to observe if the effect estimates in EAS are consistent with the ones from the EUR studies. For benchmarking, we also assessed the reproducibility of these established loci in ancestry-matched cohorts. We used independent EUR GWAS for depression with different sample sizes (Bio*Me*, BioVU, FinnGen[26], 23andMe).

Heritability and genetic correlations

We estimated the SNP heritability (h^2) for each depression phenotype in EAS (meta-analysed cohorts) using used LD score (LDSC) regression[27]. We also used bivariate GREML implemented in the GCTA software[28] to estimate h^2 for the two large Chinese datasets, CONVERGE and CKB (symptom-based definition), that contribute the majority of samples in our analysis for which genotype data were available. For this we excluded, related individuals and used hard-calls for variants with call rate>0.95 and MAF>0.01. For this analysis we used a variety of prevalence estimates, ranging from 6.5%[29] to 15%[30].

To characterise the genetic architecture of depression, we estimated genetic correlations between depression in EAS and EUR studies. For clinical depression in EUR samples, we used the summary statistics from 45,396 cases with DSM-based diagnosis of major depressive disorder and 97,250 controls from a meta-analysis of 33 independent cohorts included in the latest GWAS[17], excluding UKB and 23andMe. Additionally, we generated a symptom-based definition for EUR samples using the PHQ-9 questionnaire and a cut-off score of 10[31], yielding 6,510 affected individuals and 116,697 controls from UK Biobank[10, 32].

To assess the sharing of genetic risk factors for depression across the genome between the two populations, we estimated trans-ancestry genetic correlations using POPCORN[33]. We estimated the genetic effect correlation which compares effects independent of allele frequency differences between the two populations. LDSC was also used to estimate genetic correlations between different outcomes within each ancestry group. The default LD Scores computed using 1000 Genomes EAS data were used as a reference for the LD estimates. We also assessed the genetic overlap with other traits using publicly available summary statistics (PGC, NHGRI-EBI GWAS catalogue) from EAS and EUR populations, using LDSC and POPCORN respectively, as described above. We only present genetic correlation estimates where the standard error (SE) was less than 0.3.

To aid interpretation of the trans-ancestry genetic correlations, we also gathered estimates for other traits. We extracted genetic correlations between EUR and EAS from publications[34-37]. Additionally, we used publicly available summary statistics from Biobank Japan[38, 39] and EUR GWASs to estimate correlations for coronary artery disease (CAD)[40], breast cancer[41] and age at menarche[42] using POPCORN as outlined above.

Supplementary results

Characterisation of novel loci

Variant rs4656484 at a previously unreported locus, 1q24.1, was associated with depression with $P=4.4 \times 10^{-8}$ (beta for C allele=-0.018, SE=0.003, effect allele frequency (EAF)=0.635) (Table 1). In the UK Brain Expression Consortium resource (UKBEC)[43] rs4656484 was associated with expression of *LMX1A* (LIM Homeobox Transcription Factor 1 Alpha), which has been linked to dopamine neuron development[44]. The tissue group showing the strongest eQTL association was frontal cortex ($P=1.1 \times 10^{-4}$). In GTEx and ROSMAP[45], rs4556484 showed significant eQTL association with *FAM78B* (Family with sequence similarity 78 member B) in thyroid (GTEx, $P=7.7 \times 10^{-5}$), cortex

© 2021 Giannakopoulou O et al. *JAMA Psychiatry*.

(GTEx, P=0.046) and brain (ROSMAP, P=0.001). FAM78B forms a ribonucleoprotein complex, which shuttles RNA between the nucleus and cytosol[46].

A novel locus at 7p21.2 was associated with depression at genome-wide significance in the analysis of the East Asia based studies (Table 1). The lead SNP, rs10240457 (EAF=0.646, beta for A-allele=0.028, SE=0.005, P=5.0x10^{-9}) is intronic to *AGMO* (Alkylglycerol Monooxygenase). This gene cleaves the O-alkyl bond of ether lipids which are essential components of brain membranes and function in cell-signalling and other critical biological processes.

We carried out a meta-analysis for the broad depression outcome in EAS and the largest GWAS of depression in EUR samples[17] (Figure 1B , Supplementary Figure 4). The lead variant at 1q25.2, rs7548487, (beta for A allele= -0.013, SE=0.002, P=1.29x10^{-8}) is located in an intron of *ASTN1* (astrotactin 1). Astrotactin is a neuronal adhesion molecule required for glial-guided migration of young postmitotic neuroblasts in cortical regions of the developing brain[47]. The C-allele of the lead variant at 18q12.1, rs547488 had beta 0.008 (SE=0.001) and P=3.3x10^{-8}. It is located downstream of *CDH2* (cadherin 2) and is nominally associated with the expression of *CDH2* in the brain (UKBEC, P=0.03) and from BrainSeq[48] (P=0.027). CDH2 encodes N-cadherin, which expresses broadly in multiple tissues and has been shown to play a role in the development of the nervous system and be associated with neurodevelopmental disorders[49]. The third locus is 22q13.31 with lead variant rs12160976 (beta for A allele=-0.009, SE=0.002, P=1.6x10^{-8}).

Gene-based analysis

We also performed a gene-level aggregate test based on the meta-analysis summary statistics using MAGMA (v1.08), as implemented in FUMA[20]. The ETS Variant Transcription Factor 5 (3q27.2) gene, was the only gene that passed the significance threshold (P=6.9x10^{-6}). It has been previously associated with depression risk in an EUR study[50].

Reproducibility

In addition to the comparisons described in the main manuscript, to rule out that the low reproducibility rates are due to differences in LD patterns between the ancestry groups, we created credible sets of SNPs that are likely to contain the causal variants and assessed their associations in the EAS data. Of the 102 credible sets, 13 (12.7%) contained variant(s) with P<0.01 in the EAS association analysis with depression. We also assessed a high-confidence set of loci from the largest EUR meta-analysis that were replicated in an independent dataset of 23andMe[8]. Out of the 86 which were available in the EAS meta-analysis, 13 (15.1%) of the credible sets contained a variant with P<0.01.

© 2021 Giannakopoulou O et al. *JAMA Psychiatry*.

eFigure 1. Quantile-quantile Plot Illustrating the GWAS Meta-Analysis for Depression in 15,771 Individuals With Depression and 178,777 Controls with East Asian Ancestry

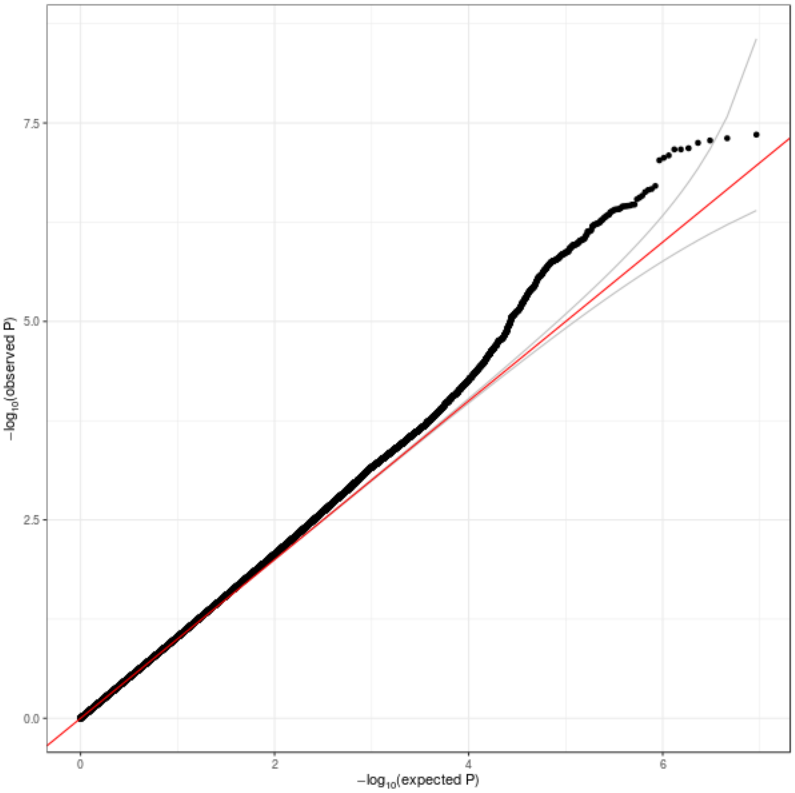

It shows the deviation of association test statistics (black dots) from the distribution expected under the null hypothesis (red line). The grey lines indicated the lower and upper 95% confidence bands. The inflation statistic was λ=1.035 and λ_{1000}=1.001 when scaled to a sample size of 1000 cases and 1000 controls (LDSC intercept 1.01 (0.01)).

© 2021 Giannakopoulou O et al. *JAMA Psychiatry*.

eFigure 2. Forest Plot for rs4656484 Which Was Genome-Wide Significant in the Depression East Asian Meta-Analysis Based on all Studies

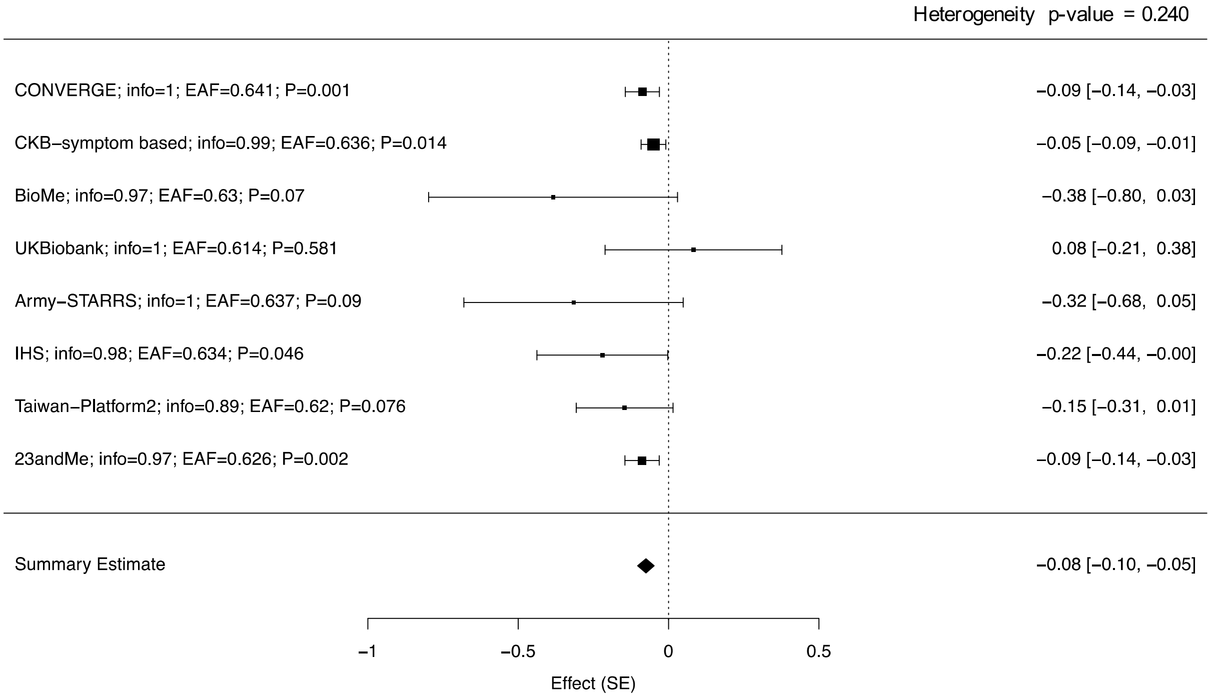

The plot shows the effect size estimates and associated confidence interval for each of the studies. The variant was not available in the Women’s Health Initiative and Taiwan-Platform 1 datasets due to insufficient imputation quality. IHS=Intern Health Study; CKB=China Kadoorie Biobank (symptom-based depression outcome). INFO=imputation accuracy score; EAF=effect allele frequency, P=p-value for the association with depression in the study.

© 2021 Giannakopoulou O et al. *JAMA Psychiatry.*

eFigure 3. Forest Plots of rs10240457 Which Was Genome-Wide Significant in the Depression Meta-Analysis of Studies Based in East Asian Countries

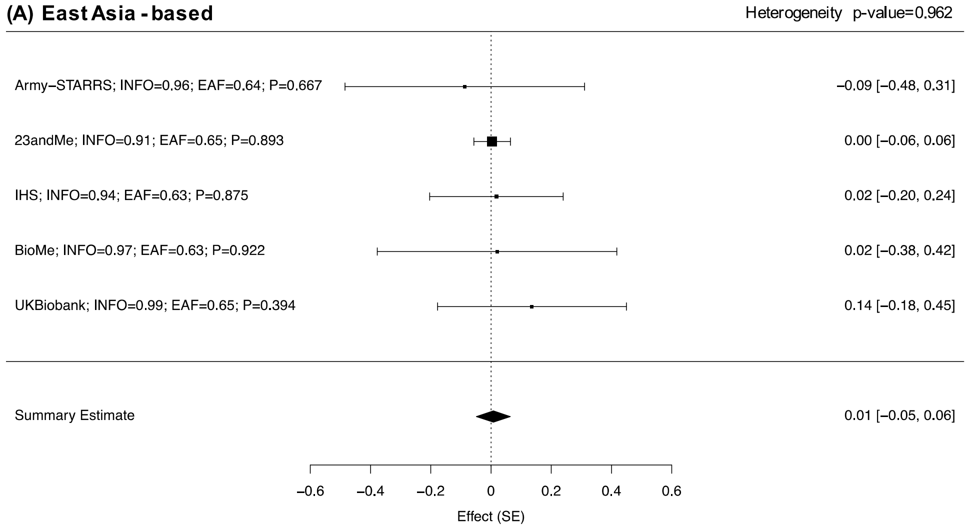

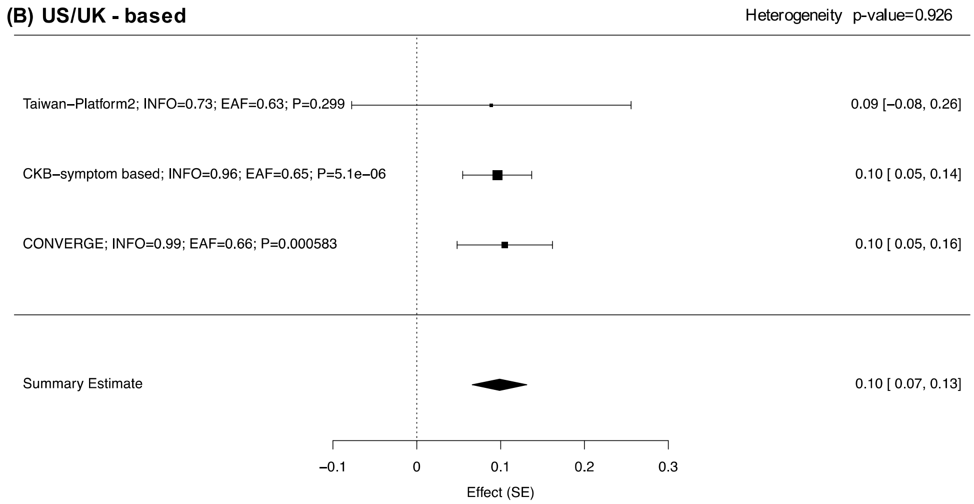

(A) effect size estimates and confidence interval for each of the studies in the East-Asia based meta-analysis and (B) results for the US/UK based studies with ancestrally East Asian samples. The variant was not available in WHI and Taiwan-Platform 1 datasets. IHS=Intern Health Study; CKB=China Kadoorie Biobank (symptom-based depression outcome).

© 2021 Giannakopoulou O et al. *JAMA Psychiatry*.

eFigure 4. Quantile-Quantile Plot Illustrating the Meta-Analysis of East Asian Results for Depression With the Largest GWAS in Europeans (Howard et al., 2019)

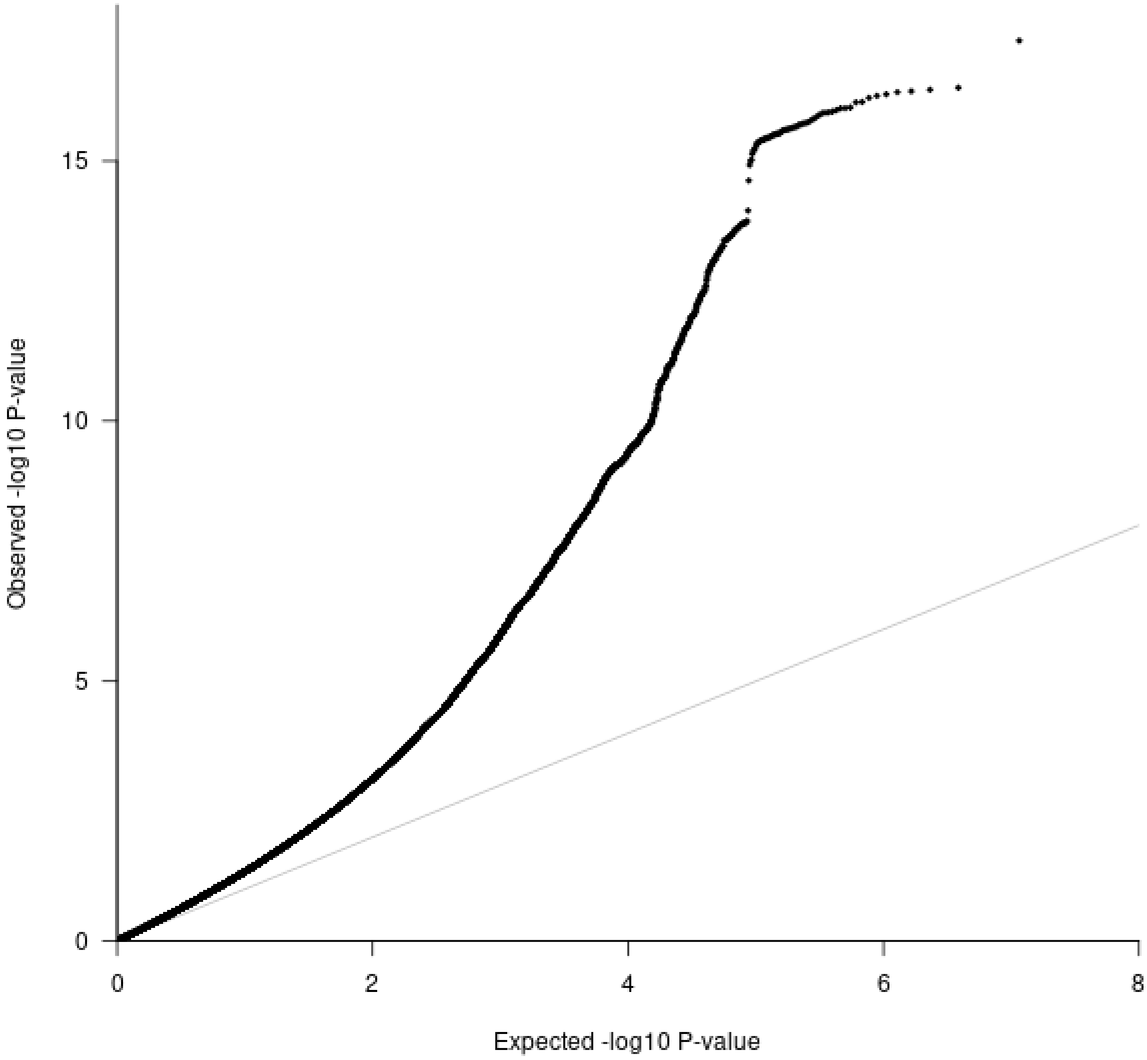

The inflation factor war λ = 1.383, λ_{1000} = 1.001.

© 2021 Giannakopoulou O et al. *JAMA Psychiatry.*

eFigure 5. Forest Plots for the Two Previously Reported Depression Loci Based on the Chinese CONVERGE Study

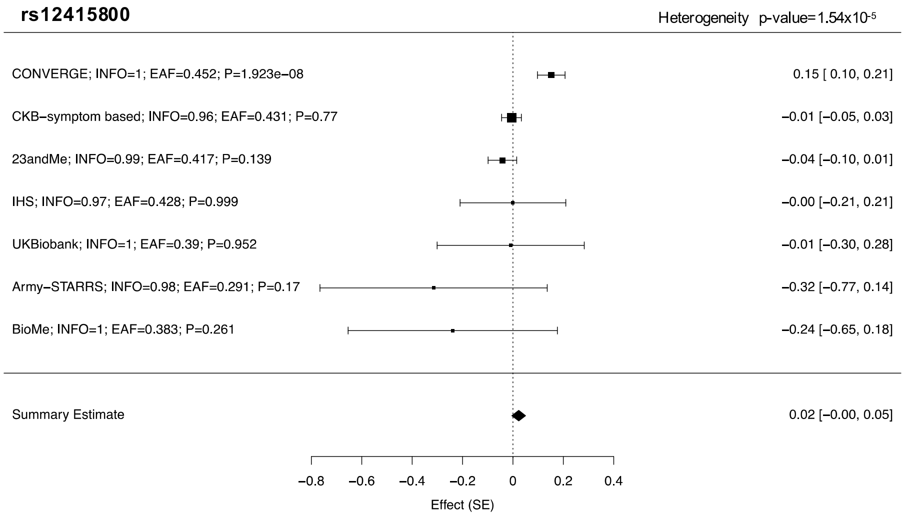

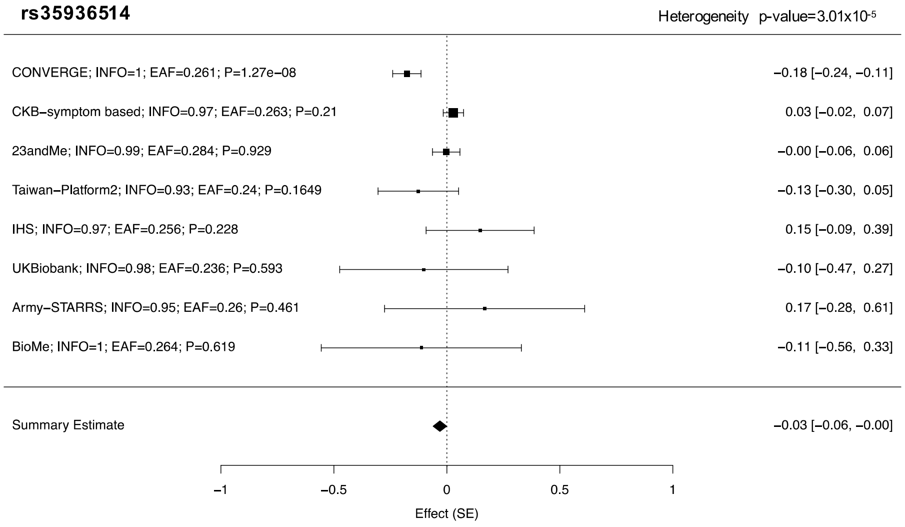

The plot shows the effect size estimates and associated confidence interval for each of the studies. IHS=Intern Health Study; CKB=China Kadoorie Biobank (symptom-based depression outcome).

© 2021 Giannakopoulou O et al. *JAMA Psychiatry*.

eFigure 6. Effect Estimates for Depression of Previously Reported Depression Loci in the Discovery European Study vs in the East Asian Meta-Analysis

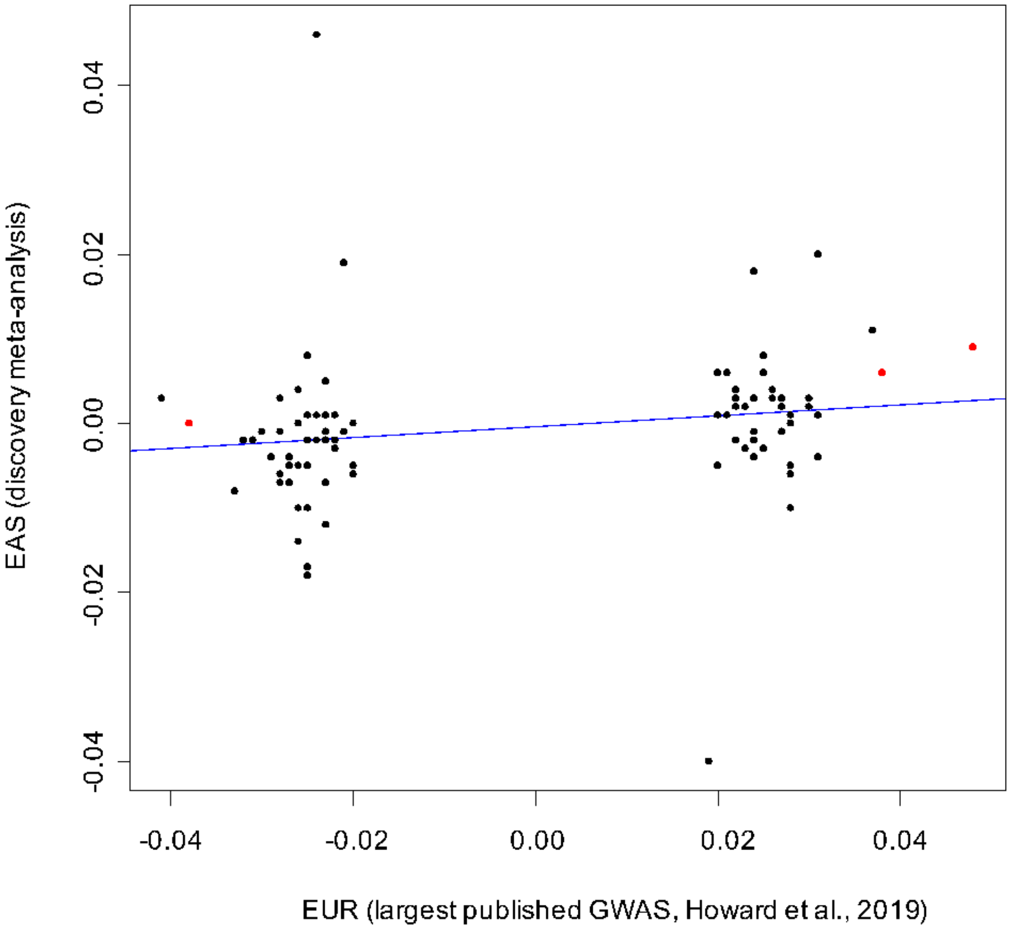

The effects are presented in log odds ratios. Variants with more than 80% power to replicate in the EAS analysis are coloured in red. The slope for regression between effect sizes is 0.065. EAS=East-Asian ancestry samples (current study), EUR=European-ancestry samples[17].

© 2021 Giannakopoulou O et al. *JAMA Psychiatry.*

eFigure 7. Genetic Correlations Between the Clinical and Symptom-Based Depression Phenotypes in East Asians and Other Traits in Europeans

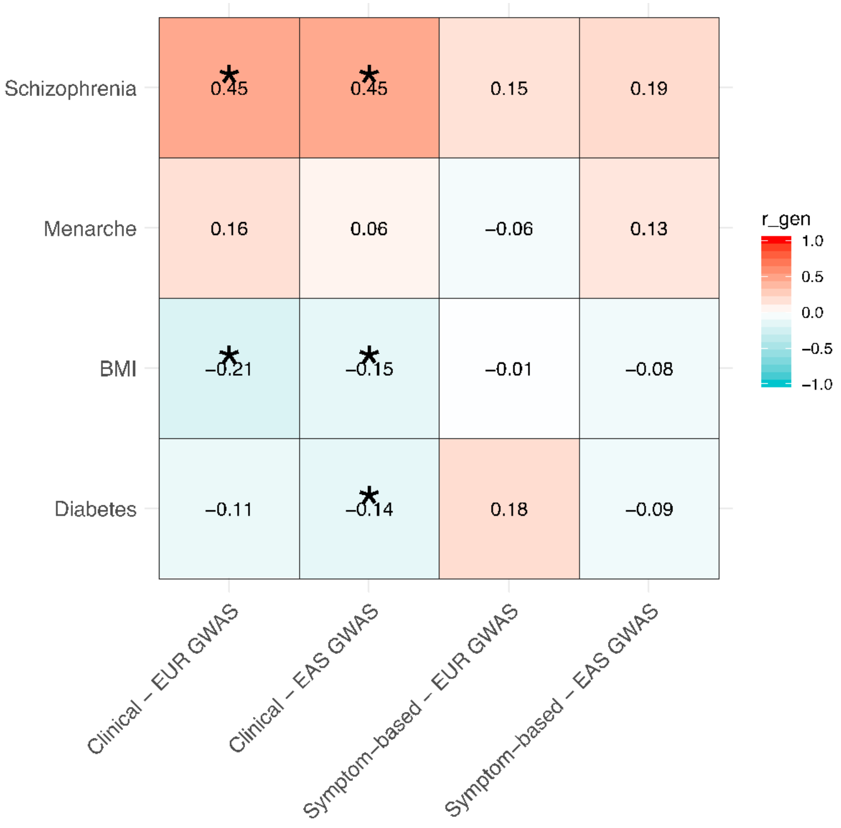

For this analysis we used published summary statistics for schizophrenia, age of menarche, body mass index (BMI) and type 2 diabetes, from European (EUR) GWAS (LDSC) and East Asian (EAS) GWASs (POPCORN). Colours correspond to direction and strength of the genetic correlations (r_{gen}). Statistically significant genetic correlations are indicated by a star (*).

© 2021 Giannakopoulou O et al. *JAMA Psychiatry.*

eTable 2. Details of the Genotyping for Each Dataset Included in the Discovery East Asian Meta-Analysis for Depression

Study	Genotyping Array	Imputation panel	Inflation factor (λ)	N cases	N controls	N markers (included in the meta-analysis)
CKB	Custom-designed Affymetrix	1000Genomes	1.000	5376	69998	10,834,708
CONVERGE	Low-depth Whole Genome Sequencing	1000Genomes Phase 1 Asian panel	1.075	5303	5337	5,987,610
23andMe	Illumina HumanHap550+ BeadChip, Illumina OmniExpress+ BeadChip, a custom-designed array and Illumina Infinium Global Screening Array	1000 Genomes Phase 3 haplotypes with the UK10 imputation reference panel	1.024	2729	90310	9,072,919
Taiwan MDD study - Platform 1	Affymetrix CHB Array, Affymetrix TWB1.0 Array, Illumina Human Omni Express Exiome Beadchip*	1000Genomes Phase 3	0.945	988	6075	551,251
Taiwan MDD study - Platform 2	Affymetrix TWB2.0 Array	1000Genomes Phase 3	1.013	360	2317	4,390,026
IHS	CoreExome	Haplotype Reference Consortium panel	1.023	294	544	4,626,568
WHI	CardioMetabochip	-	1.045	454	2553	94,527
UKB	UKBB Axiom & BiLEVE	1000Genomes Phase 3 + Haplotype Reference Consortium	1.039	133	366	8,037,956
Army STARRS	Illumina OmniExpress + Exome array	1000Genomes	1.06	74	442	6,591,199
Bio*Me*	Infinium Global Screening Array-24 v2.0	1000Genomes Phase 3	0.995	60	835	6,336,224
**Meta-analysis**	**-**	**-**		**15,771**	**178,777**	

*Due to the differences in genotyping arrays, imputation and meta-analysis was performed separately. Samples genotyped with Affymetrix TWB2.0 array were considered a separate dataset, while the other samples were combined in a different set. CKB=China Kadoorie Biobank; MDD=Major Depressive Disorder; IHS=Intern Health Study; WHI=Women's Health Initiative; UKB=UK Biobank

© 2021 Giannakopoulou O et al. *JAMA Psychiatry*.

eTable 7. The Two Genome-Wide Significant Depression Loci in CONVERGE and the Relevant Results in the Other East Asian Ancestry Datasets of the Current Study and the Two Largest European Studies

**rs12415800**								
**Study**	**EA**	**OA**	**EAF**	**BETA**	**SE**	**P**	**Ncases/ Ncontrols**	**Power**
CONVERGE	A	G	0.452	0.152	0.028	0.00000001923	5303/5337	0.127
CKB-Symptom based	A	G	0.431	-0.006	0.020	0.77	5376/69998	1.000
CKB-Lifetime diagnosis	A	G	0.431	-0.024	0.041	0.55	1305/69998	0.942
23andMe	A	G	0.417	-0.042	0.029	0.139	2729/90310	0.999
Taiwan MDD Study - Platform 1	NA	NA	NA	NA	NA	NA	988/6075	NA
Taiwan MDD Study - Platform 1	NA	NA	NA	NA	NA	NA	360/2317	NA
WHI	NA	NA	NA	NA	NA	NA	454/2553	NA
IHS	A	G	0.428	-0.0001	0.107	0.999	294/544	0.292
UK Biobank	A	G	0.390	-0.009	0.149	0.952	133/366	0.179
Army STARRS	A	G	0.291	-0.315	0.230	0.170	74/442	0.119
BioMe	A	G	0.383	-0.239	0.212	0.261	60/835	0.115
PGC Major Depression Study (Wray et al., 2018)*	A	G	0.027	-0.004	0.039	0.797	59851/113154	1.000

© 2021 Giannakopoulou O et al. *JAMA Psychiatry*.

Depression (Howard et al., 2019)*	A	G	0.016	-0.004	0.019	0.843	179756/329443	1.000
**rs35936514**								
**Study**	**EA**	**OA**	**EAF**	**BETA**	**SE**	**P**	**Ncases/Ncontrols**	**Power**
CONVERGE	T	C	0.261	-0.177	0.032	0.0000000127	5303/5337	0.142
CKB-Symptom based	T	C	0.263	0.029	0.023	0.21	5376/69998	1.000
CKB-Lifetime diagnosis	T	C	0.263	0.063	0.046	0.17	1305/69998	0.972
23andMe	T	C	0.284	-0.003	0.031	0.929	2729/90310	1.000
Taiwan MDD Study - Platform 1	NA	NA	NA	NA	NA	NA	988/6075	NA
Taiwan MDD Study - Platform 1	T	C	0.240	-0.126	0.091	0.1649	360/2317	0.495
WHI	NA	NA	NA	NA	NA	NA	454/2553	NA
IHS	T	C	0.256	0.147	0.122	0.228	294/544	0.334
UK Biobank	T	C	0.236	-0.102	0.190	0.593	133/366	0.199
Army STARRS	T	C	0.260	0.167	0.226	0.461	74/442	0.148
BioMe	T	C	0.264	-0.113	0.226	0.619	60/835	0.132
PGC Major Depression Study (Wray et al., 2018)*	T	C	0.057	-0.009	0.019	0.293	59851/113154	1.000
Depression (Howard et al., 2019)*	T	C	0.048	-0.010	0.011	0.356	179756/329443	1.000

The effect sizes (beta coefficients) are reported for the effect allele. The power calculations are based on the natural log of the combined Odds Ratios in the discovery study (Cai et al., 2015), the EAF of each variant in each individual study, as well as the sample size of each study. The * denotes the studies conducted in studies with European ancestry samples.
EA=Effect Allele; OA=Other Allele; EAF=EA frequency; SE=Standard Error; CKB=China Kadoorie Biobank; MDD=Major Depressive Disorder; WHI=Women's Health Initiative; IHS=Intern Health Study; PGC=Psychiatric Genetics Consortium

© 2021 Giannakopoulou O et al. *JAMA Psychiatry*.

eTable 9. Reproducibility of Established Depression Loci From Howard et al.,2019 in Independent Samples of European Ancestry and in the East Asian Depression GWAS

Cohort Name	Ancestry	Phenotype definition	N cases	N controls	N variants	Observed (%)	Expected (%)	Observed /expected
BioMe	EUR	EHR	1,456	8,304	102	6.86%	9.28%	0.74
BioVU	EUR	HER	7,757	24,723	71	18.31%	28.17%	0.65
FinnGen	EUR	EHR	17,794	156,611	92	21.74%	55%	0.40
23andMe	EUR	self-reported	105,114	1,757,384	96	84.38%	99.79%	0.86
EAS GWAS	EAS	broad	15,771	178,777	94	10.6%	42.5%	0.25

*N variants: number of lead variants from genome-wide significant loci in Howard et al which were present in the look up study
EUR: European ancestry samples
EAS: East Asian ancestry samples
EHR: Medical diagnosis of major depressive disorder from Electronic Healthcare Records

© 2021 Giannakopoulou O et al. *JAMA Psychiatry*.

eTable 10. SNP-Heritabilities for the Different Depression-Definition Outcomes Considered in This Study

Study	Observed h^2 (SE)	Liability scale h^2 (SE)		
		K=6.5%	K=12%	K=15%
Broad depression, EAS meta-analysis	0.009 (0.002)	2.9% (0.01)	3.5% (0.01)	3.8% (0.01)
Clinical depression, EAS meta-analysis	0.024 (0.006)	6.7% (0.02)	8.2% (0.02)	8.8% (0.02)
Symtom-based depression, EAS meta-analysis	0.012 (0.006)	3.8% (0.02)	4.6% (0.03)	4.6% (0.02)
CONVERGE	0.284 (0.032)	26.2% (0.03)	31.7% (0.04)	34.1% (0.04)
China Kadoorie Biobank (symptom-based definition)	0.045 (0.017)	6.4% (0.02)	7.8% (0.03)	8.4% (0.03)
Clinical depression, EUR meta-analysis (PGC)	0.073 (0.005)	7.4% (0.01)	9% (0.01)	9.7% (0.01)
Symptom-based depression, UK Biobank European dataset (PHQ9)	0.028 (0.004)	12.2% (0.02)	14.8% (0.02)	15.9% (0.02)

EAS=East Asian ancestry; SE=Standard error

© 2021 Giannakopoulou O et al. *JAMA Psychiatry*.

eTable 11. Transancestry Genetic Correlation Estimates for Diverse Phenotypes Between Samples With East Asian and European Ancestry

**Trait**	**PMID**	**N EAS (cases) a**	**N EUR (cases)**	**Country b**	**Genetic correlation**	**Standard error**	***P* value c**
Schizophrenia	31740837	58,140 (22,778)	82,315 (35,476)	Multiple	0.98	0.03	>0.05
Schizophrenia	30285260	9,348 (1,940)	77,096 (33,640)	Japan	0.577	-	1.83*10-13
Bipolar disorder	30285260	64,851 (2,964)	16,731 (7,481)	Japan	0.718	-	3.65*10-3
Smoking initiation	31089300	165,436 (83,810)	359,751(297,127)	Japan	0.717	0.035	2.2*10-16
HDL cholesterol	31551420	162,255	188,577	Japan	0.999	0.081	
HDL cholesterol	31551420	21,295	188,577	China	0.999	-	
LDL cholesterol	31551420	162,255	188,577	Japan	0.959	0.138	
LDL cholesterol	31551420	21,295	188,577	China	0.778	0.300	
Triglyceride levels	31551420	162,255	188,577	Japan	0.961	0.066	
Triglyceride levels	31551420	21,295	188,577	China	0.999	-	
Coronary artery disease	32514122 (EAS), 28714975 (EUR)	212,453 (29,319)	332,477 (71,602)	Japan	0.908	0.083	0.27
Breast cancer	32514122 (EAS), 29059683 (EUR)	95,283 (5,552)	119,014 (69,980)	Japan	0.761	0.196	0.22
Age at menarche	29773799 (EAS), 28436984 (EUR)	67,029	252,000	Japan	0.0801	0.0688	0.0036

a. Number of East Asians (EAS) in calculation with number of Europeans (EUR) in brackets; b. Country where the East Asian samples were collected; c. P value for whether the genetic correlation is different from 1.

© 2021 Giannakopoulou O et al. *JAMA Psychiatry*.

eTable 12. Transancestry Genetic Correlations of Clinical and Symptom-Based Depression in East Asian Datasets With Other Traits, Based on European Studies

Trait	PMID	Genetic correlation	Standard error	Confidence Intervals
		Clinical depression		
Body mass index (BMI)*	30124842	-0.212	0.084	-0.378, -0.047
Coronary artery disease (CAD)	28714975	-0.253	0.160	-0.567, 0.06
Age of menarche	28436984	0.161	0.099	-0.033, 0.356
Smoking (ever vs never)	30643251	-0.156	0.089	-0.331, 0.019
Subjective well-being	27089181	-0.393	0.198	-0.781, -0.004
Type 2 diabetes (T2D)	30054458	-0.113	0.113	-0.334, 0.108
Alcohol dependence (AD)	30482948	-0.045	0.298	-0.629, 0.54
Alcohol consumption (drinks per week)	30643251	-0.022	0.110	-0.237, 0.194
Attention-Deficit/Hyperactivity Disorder (ADHD)	30478444	-0.063	0.150	-0.356, 0.231
Alzheimer’s disease	24418058	NA	NA	NA
Anorexia nervosa*	31308545	0.502	0.158	0.193, 0.811
Anxiety	26754954	NA	NA	NA
Autism	30804558	0.130	0.171	-0.205, 0.465
Bipolar disorder*	31043756	0.710	0.153	0.41, 1.009
Neuroticism	27089181	0.303	0.150	0.009, 0.596
Schizophrenia*	31740837	0.449	0.109	0.234, 0.664
Depressive symptoms	27089181	0.311	0.212	-0.103, 0.726
Symptom-based depression (UKB)	30305743	0.223	0.181	-0.131, 0.577
Clinical depression (PGC)*	29700475	0.413	0.159	0.101, 0.725

© 2021 Giannakopoulou O et al. *JAMA Psychiatry*.

		Symptom-based depression		
Body mass index (BMI)	30124842	-0.009	0.100	-0.206, 0.187
Coronary artery disease (CAD)	28714975	0.170	0.158	-0.14, 0.479
Age of menarche	28436984	-0.065	0.106	-0.273, 0.144
Smoking (ever vs never)	30643251	-0.172	0.126	-0.419, 0.075
Alcohol dependence (AD)	30482948	NA	NA	NA
Alcohol consumption (drinks per week)	30643251	-0.002	0.140	-0.276, 0.273
Attention-Deficit/Hyperactivity Disorder (ADHD)	30478444	-0.115	0.148	-0.404, 0.175
Alzheimer's disease	24418058	NA	NA	NA
Anorexia nervosa*	31308545	0.449	0.193	0.07, 0.827
Anxiety	26754954	NA	NA	NA
Autism	30804558	0.182	0.219	-0.247, 0.611
Bipolar disorder	31043756	0.072	0.161	-0.243, 0.388
Neuroticism	27089181	0.840	0.216	0.417, 1.263
Schizophrenia	31740837	0.153	0.142	-0.126, 0.431
Subjective well-being*	27089181	-0.502	0.195	-0.885, -0.119
Type 2 diabetes (T2D)	30054458	0.177	0.166	-0.148, 0.503
Depressive symptoms	27089181	NA	NA	NA
Symptom-based depression (UKB)	30305743	0.433	0.281	-0.118, 0.985
Clinical depression (PGC)	29700475	0.558	0.221	0.124, 0.992

The * denotes the significance, based on the confidence intervals. The traits that did not provide robust results (Standard Error > 0.3) are not presented (Not Available (NA)).

© 2021 Giannakopoulou O et al. *JAMA Psychiatry*.

eTable 13. Genetic Correlations of Clinical and Symptom-Based Depression in European Datasets With Other Traits

Trait	PMID	rg	SE	Z	P-value
		Clinical depression - Psychiatric Genetics Consortium			
Symptom-based depression	30305743	0.814	0.080	10.168	2.77E-24
Attention-Deficit/Hyperactivity Disorder (ADHD)	30478444	0.570	0.040	14.139	2.19E-45
Alcohol dependence (AD)	30482948	0.583	0.119	4.915	8.89E-07
Alzheimer's disease	24418058	0.041	0.079	0.515	6.07E-01
Anorexia nervosa	31308545	0.305	0.047	6.473	9.60E-11
Anxiety	26754954	0.855	0.168	5.094	3.51E-07
Autism	30804558	0.417	0.047	8.918	4.76E-19
Bipolar disorder	31043756	0.345	0.038	9.129	6.95E-20
Alcohol consumption (drinks per week)	30643251	0.090	0.036	2.511	1.20E-02
Depressive symptoms	27089181	0.964	0.043	22.342	1.45E-110
Neuroticism	27089181	0.720	0.046	15.712	1.25E-55
Schizophrenia	31740837	0.349	0.030	11.772	5.44E-32
Age at menarche	28436984	-0.091	0.031	-2.902	3.70E-03
Body Mass Index (BMI)	30124842	0.111	0.025	4.368	1.26E-05
Smoking (ever vs never)	30643251	0.321	0.032	10.156	3.10E-24
Subjective wellbeing	27089181	-0.697	0.053	-13.092	3.67E-39
Type 2 diabetes (T2D)	30054458	0.185	0.031	5.926	3.11E-09
Coronary Artery Disease (CAD)	28714975	0.203	0.037	5.463	4.68E-08

© 2021 Giannakopoulou O et al. *JAMA Psychiatry*.

Trait	PMID	rg	SE	Z	P-value
		**Clinical depression - Psychiatric Genetics Consortium**			
Broad depression (self-reported help-seeking behaviour for mental health difficulties or diagnosis of a depressive mood disorder from linked hospital admission records)	29662059	0.878	0.038	23.160	1.17E-118
ICD-coded MDD (hospital admission diagnosis)	29662059	0.907	0.070	13.021	9.26E-39
Probable MDD (cardinal symptoms + broad depression)	29662059	0.840	0.085	9.925	3.26E-23
Depression	30718901	0.946	0.022	43.498	0
		**Symptom-based depression - UK Biobank**			
Clinical depression	29700475	0.814	0.080	10.168	2.77E-24
Attention-Deficit/Hyperactivity Disorder (ADHD)	30478444	0.520	0.070	7.388	1.49E-13
Alcohol dependence (AD)	30482948	0.375	0.140	2.682	7.31E-03
Alzheimer's disease	24418058	-0.065	0.108	-0.601	5.48E-01
Anorexia nervosa	31308545	0.032	0.073	0.441	6.59E-01
Anxiety	26754954	0.596	0.211	2.826	4.72E-03
Autism	30804558	0.315	0.097	3.263	1.10E-03
Bipolar disorder	31043756	0.118	0.055	2.155	3.12E-02
Alcohol consumption (drinks per week)	30643251	-0.063	0.053	-1.202	2.29E-01
Depressive symptoms	27089181	0.894	0.091	9.873	5.47E-23
Neuroticism	27089181	0.608	0.095	6.427	1.30E-10

© 2021 Giannakopoulou O et al. *JAMA Psychiatry*.

Trait	PMID	rg	SE	Z	P-value
		**Symptom-based depression - UK Biobank**			
Schizophrenia	31740837	0.218	0.049	4.481	7.44E-06
Age at menarche	28436984	-0.051	0.039	-1.314	1.89E-01
Body Mass Index (BMI)	30124842	0.314	0.041	7.700	1.36E-14
Smoking (ever vs never)	30643251	0.290	0.044	6.683	2.34E-11
Subjective wellbeing	27089181	-0.750	0.086	-8.722	2.73E-18
Type 2 diabetes (T2D)	30054458	0.353	0.063	5.603	2.11E-08
Coronary Artery Disease (CAD)	28714975	0.310	0.057	5.464	4.67E-08
Broad depression (self-reported help-seeking behaviour for mental health difficulties or diagnosis of a depressive mood disorder from linked hospital admission records)	29662059	0.681	0.061	11.254	2.22E-29
ICD-coded MDD (hospital admission diagnosis)	29662059	0.817	0.109	7.521	5.44E-14
Probable MDD (cardinal symptoms + broad depression)	29662059	0.724	0.103	7.019	2.23E-12
Depression	30718901	0.708	0.059	11.925	0

rg= genetic correlation; SE= standard error

© 2021 Giannakopoulou O et al. *JAMA Psychiatry*.

eTable 14. Genetic Correlations of Clinical and Symptom-Based Depression in East Asian Datasets With Other Traits, Based on East Asian Studies

Trait	PMID	rg	SE	Z	P-value
		**Clinical depression**			
Schizophrenia	31740837	0.447	0.085	5.256	1.47E-07
Type 2 diabetes (T2D)	30718926	-0.143	0.072	-1.997	0.046
Body Mass Index (BMI)	28892062	-0.147	0.061	-2.402	0.016
Age at menarche	29773799	0.060	0.087	0.687	0.492
High-density lipoprotein (HDL)	28334899	0.093	0.154	0.605	0.545
Low-density lipoprotein (LDL)	28334899	0.177	0.134	1.323	0.186
Triglycerides (TG)	28334899	-0.073	0.165	-0.441	0.660
Total cholesterol (TC)	28334899	0.127	0.116	1.097	0.273
		**Symptom-based depression**			
Schizophrenia	31740837	0.189	0.137	1.380	0.168
Type 2 diabetes (T2D)	30718926	-0.088	0.120	-0.734	0.463
Body Mass Index (BMI)	28892062	-0.082	0.098	-0.832	0.405
Age at menarche	29773799	0.130	0.134	0.971	0.332
High-density lipoprotein (HDL)	28334899	0.145	0.219	0.660	0.509
Low-density lipoprotein (LDL)	28334899	0.133	0.226	0.587	0.557
Triglycerides (TG)	28334899	-0.321	0.235	-1.368	0.172
Total cholesterol (TC)	28334899	0.085	0.208	0.411	0.681

rg= genetic correlation; SE= standard error

© 2021 Giannakopoulou O et al. *JAMA Psychiatry*.

eReferences

1. Chen Z, Chen J, Collins R, et al. China Kadoorie Biobank of 0.5 million people: survey methods, baseline characteristics and long-term follow-up. *Int J Epidemiol*. Dec 2011;40(6):1652-66. doi:10.1093/ije/dyr120
2. Wojcik GL, Graff M, Nishimura KK, et al. Genetic analyses of diverse populations improves discovery for complex traits. *Nature*. 06 2019;570(7762):514-518. doi:10.1038/s41586-019-1310-4
3. CONVERGE Consortium. Sparse whole-genome sequencing identifies two loci for major depressive disorder. *Nature*. Jul 2015;523(7562):588-91. doi:10.1038/nature14659
4. Chen CH, Yang JH, Chiang CWK, et al. Population structure of Han Chinese in the modern Taiwanese population based on 10,000 participants in the Taiwan Biobank project. *Hum Mol Genet*. 12 2016;25(24):5321-5331. doi:10.1093/hmg/ddw346
5. WHI Study Group. Design of the Women's Health Initiative clinical trial and observational study. The Women's Health Initiative Study Group. *Control Clin Trials*. Feb 1998;19(1):61-109. doi:10.1016/s0197-2456(97)00078-0
6. Wassertheil-Smoller S, Shumaker S, Ockene J, et al. Depression and cardiovascular sequelae in postmenopausal women. The Women's Health Initiative (WHI). *Arch Intern Med*. Feb 2004;164(3):289-98. doi:10.1001/archinte.164.3.289
7. Fang Y, Scott L, Song P, Burmeister M, Sen S. Genomic prediction of depression risk and resilience under stress. *Nat Hum Behav*. Jan 2020;4(1):111-118. doi:10.1038/s41562-019-0759-3
8. Levis B, Benedetti A, Thombs BD, Collaboration DSDD. Accuracy of Patient Health Questionnaire-9 (PHQ-9) for screening to detect major depression: individual participant data meta-analysis. *BMJ*. 04 2019;365:l1476. doi:10.1136/bmj.l1476
9. Lam M, Awasthi S, Watson HJ, et al. RICOPILI: Rapid Imputation for COnsortias PIpeLIne. *Bioinformatics*. Feb 2020;36(3):930-933. doi:10.1093/bioinformatics/btz633
10. Bycroft C, Freeman C, Petkova D, et al. The UK Biobank resource with deep phenotyping and genomic data. *Nature*. 10 2018;562(7726):203-209. doi:10.1038/s41586-018-0579-z
11. Conomos MP, Miller MB, Thornton TA. Robust inference of population structure for ancestry prediction and correction of stratification in the presence of relatedness. *Genet Epidemiol*. May 2015;39(4):276-93. doi:10.1002/gepi.21896
12. Ursano RJ, Colpe LJ, Heeringa SG, et al. The Army study to assess risk and resilience in servicemembers (Army STARRS). *Psychiatry*. 2014;77(2):107-19. doi:10.1521/psyc.2014.77.2.107
13. Kessler RC, Colpe LJ, Fullerton CS, et al. Design of the Army Study to Assess Risk and Resilience in Servicemembers (Army STARRS). *Int J Methods Psychiatr Res*. Dec 2013;22(4):267-75. doi:10.1002/mpr.1401
14. Stein MB, Ware EB, Mitchell C, et al. Genomewide association studies of suicide attempts in US soldiers. *Am J Med Genet B Neuropsychiatr Genet*. Dec 2017;174(8):786-797. doi:10.1002/ajmg.b.32594
15. Belbin GM, Odgis J, Sorokin EP, et al. Genetic identification of a common collagen disease in puerto ricans via identity-by-descent mapping in a health system. *Elife*. 09 2017;6doi:10.7554/eLife.25060
16. Tung JY, Do CB, Hinds DA, et al. Efficient replication of over 180 genetic associations with self-reported medical data. *PLoS One*. 2011;6(8):e23473. doi:10.1371/journal.pone.0023473
17. Howard DM, Adams MJ, Clarke TK, et al. Genome-wide meta-analysis of depression identifies 102 independent variants and highlights the importance of the prefrontal brain regions. *Nat Neurosci*. 03 2019;22(3):343-352. doi:10.1038/s41593-018-0326-7
18. Zhu Z, Zhang F, Hu H, et al. Integration of summary data from GWAS and eQTL studies predicts complex trait gene targets. *Nat Genet*. 05 2016;48(5):481-7. doi:10.1038/ng.3538
19. Watanabe K, Taskesen E, van Bochoven A, Posthuma D. Functional mapping and annotation of genetic associations with FUMA. *Nat Commun*. 11 2017;8(1):1826. doi:10.1038/s41467-017-01261-5
20. de Leeuw CA, Mooij JM, Heskes T, Posthuma D. MAGMA: generalized gene-set analysis of GWAS data. *PLoS Comput Biol*. Apr 2015;11(4):e1004219. doi:10.1371/journal.pcbi.1004219
21. Ward LD, Kellis M. HaploReg: a resource for exploring chromatin states, conservation, and regulatory motif alterations within sets of genetically linked variants. *Nucleic Acids Res*. Jan 2012;40(Database issue):D930-4. doi:10.1093/nar/gkr917
22. Carvalho-Silva D, Pierleoni A, Pignatelli M, et al. Open Targets Platform: new developments and updates two years on. *Nucleic Acids Res*. 01 2019;47(D1):D1056-D1065. doi:10.1093/nar/gky1133
23. Staley JR, Blackshaw J, Kamat MA, et al. PhenoScanner: a database of human genotype-phenotype associations. *Bioinformatics*. 10 2016;32(20):3207-3209. doi:10.1093/bioinformatics/btw373

© 2021 Giannakopoulou O et al. *JAMA Psychiatry*.

24. MacArthur J, Bowler E, Cerezo M, et al. The new NHGRI-EBI Catalog of published genome-wide association studies (GWAS Catalog). *Nucleic Acids Res*. 01 2017;45(D1):D896-D901. doi:10.1093/nar/gkw1133
25. Sham PC, Purcell SM. Statistical power and significance testing in large-scale genetic studies. *Nat Rev Genet*. May 2014;15(5):335-46. doi:10.1038/nrg3706
26. Tabassum R, Rämö JT, Ripatti P, et al. Genetic architecture of human plasma lipidome and its link to cardiovascular disease. *Nat Commun*. 09 2019;10(1):4329. doi:10.1038/s41467-019-11954-8
27. Bulik-Sullivan BK, Loh PR, Finucane HK, et al. LD Score regression distinguishes confounding from polygenicity in genome-wide association studies. *Nat Genet*. Mar 2015;47(3):291-5. doi:10.1038/ng.3211
28. Yang J, Lee SH, Goddard ME, Visscher PM. GCTA: a tool for genome-wide complex trait analysis. *Am J Hum Genet*. Jan 2011;88(1):76-82. doi:10.1016/j.ajhg.2010.11.011
29. Bromet E, Andrade LH, Hwang I, et al. Cross-national epidemiology of DSM-IV major depressive episode. *BMC Med*. Jul 2011;9:90. doi:10.1186/1741-7015-9-90
30. Wray NR, Ripke S, Mattheisen M, et al. Genome-wide association analyses identify 44 risk variants and refine the genetic architecture of major depression. *Nat Genet*. 05 2018;50(5):668-681. doi:10.1038/s41588-018-0090-3
31. Manea L, Gilbody S, McMillan D. Optimal cut-off score for diagnosing depression with the Patient Health Questionnaire (PHQ-9): a meta-analysis. *CMAJ*. Feb 2012;184(3):E191-6. doi:10.1503/cmaj.110829
32. Davis KAS, Coleman JRI, Adams M, et al. Mental health in UK Biobank - development, implementation and results from an online questionnaire completed by 157 366 participants: a reanalysis. *BJPsych Open*. Feb 2020;6(2):e18. doi:10.1192/bjo.2019.100
33. Brown BC, Ye CJ, Price AL, Zaitlen N, Consortium AGENTD. Transethnic Genetic-Correlation Estimates from Summary Statistics. *Am J Hum Genet*. 07 2016;99(1):76-88. doi:10.1016/j.ajhg.2016.05.001
34. Lam M, Chen CY, Li Z, et al. Comparative genetic architectures of schizophrenia in East Asian and European populations. *Nat Genet*. 12 2019;51(12):1670-1678. doi:10.1038/s41588-019-0512-x
35. Ikeda M, Takahashi A, Kamatani Y, et al. Genome-Wide Association Study Detected Novel Susceptibility Genes for Schizophrenia and Shared Trans-Populations/Diseases Genetic Effect. *Schizophr Bull*. 06 2019;45(4):824-834. doi:10.1093/schbul/sby140
36. Matoba N, Akiyama M, Ishigaki K, et al. GWAS of smoking behaviour in 165,436 Japanese people reveals seven new loci and shared genetic architecture. *Nat Hum Behav*. 05 2019;3(5):471-477. doi:10.1038/s41562-019-0557-y
37. Kuchenbaecker K, Telkar N, Reiker T, et al. The transferability of lipid loci across African, Asian and European cohorts. *Nat Commun*. 09 2019;10(1):4330. doi:10.1038/s41467-019-12026-7
38. Horikoshi M, Day FR, Akiyama M, et al. Elucidating the genetic architecture of reproductive ageing in the Japanese population. *Nat Commun*. 05 2018;9(1):1977. doi:10.1038/s41467-018-04398-z
39. Ishigaki K, Akiyama M, Kanai M, et al. Large-scale genome-wide association study in a Japanese population identifies novel susceptibility loci across different diseases. *Nat Genet*. 07 2020;52(7):669-679. doi:10.1038/s41588-020-0640-3
40. Nelson CP, Goel A, Butterworth AS, et al. Association analyses based on false discovery rate implicate new loci for coronary artery disease. *Nat Genet*. Sep 2017;49(9):1385-1391. doi:10.1038/ng.3913
41. Michailidou K, Lindström S, Dennis J, et al. Association analysis identifies 65 new breast cancer risk loci. *Nature*. 11 2017;551(7678):92-94. doi:10.1038/nature24284
42. Day FR, Thompson DJ, Helgason H, et al. Genomic analyses identify hundreds of variants associated with age at menarche and support a role for puberty timing in cancer risk. *Nat Genet*. Jun 2017;49(6):834-841. doi:10.1038/ng.3841
43. Ramasamy A, Trabzuni D, Guelfi S, et al. Genetic variability in the regulation of gene expression in ten regions of the human brain. *Nat Neurosci*. Oct 2014;17(10):1418-1428. doi:10.1038/nn.3801
44. Hong S, Chung S, Leung K, Hwang I, Moon J, Kim KS. Functional roles of Nurr1, Pitx3, and Lmx1a in neurogenesis and phenotype specification of dopamine neurons during in vitro differentiation of embryonic stem cells. *Stem Cells Dev*. Mar 2014;23(5):477-87. doi:10.1089/scd.2013.0406
45. Bennett DA, Schneider JA, Arvanitakis Z, Wilson RS. Overview and findings from the religious orders study. *Curr Alzheimer Res*. Jul 2012;9(6):628-45. doi:10.2174/156720512801322573
46. Pérez-González A, Pazo A, Navajas R, Ciordia S, Rodriguez-Frandsen A, Nieto A. hCLE/C14orf166 associates with DDX1-HSPC117-FAM98B in a novel transcription-dependent shuttling RNA-transporting complex. *PLoS One*. 2014;9(3):e90957. doi:10.1371/journal.pone.0090957

© 2021 Giannakopoulou O et al. *JAMA Psychiatry*.

47. Fink JM, Hirsch BA, Zheng C, Dietz G, Hatten ME, Ross ME. Astrotactin (ASTN), a gene for glial-guided neuronal migration, maps to human chromosome 1q25.2. *Genomics*. Feb 1997;40(1):202-5. doi:10.1006/geno.1996.4538
48. drweinberger@libd.org BAHBGCEa, Consortium BAHBG. BrainSeq: Neurogenomics to Drive Novel Target Discovery for Neuropsychiatric Disorders. *Neuron*. Dec 2015;88(6):1078-1083. doi:10.1016/j.neuron.2015.10.047
49. Accogli A, Calabretta S, St-Onge J, et al. De Novo Pathogenic Variants in N-cadherin Cause a Syndromic Neurodevelopmental Disorder with Corpus Collosum, Axon, Cardiac, Ocular, and Genital Defects. *Am J Hum Genet*. 10 2019;105(4):854-868. doi:10.1016/j.ajhg.2019.09.005
50. Lewis CM, Ng MY, Butler AW, et al. Genome-wide association study of major recurrent depression in the U.K. population. *Am J Psychiatry*. Aug 2010;167(8):949-57. doi:10.1176/appi.ajp.2010.09091380

© 2021 Giannakopoulou O et al. *JAMA Psychiatry*.
