# Supplementary material for: The Genetic Architecture of Depression in Individuals of East Asian Ancestry: A Genome-Wide Association Study
Source: JAMA Psychiatry. 2021 Sep 29;78(11):1–12. doi: 10.1001/jamapsychiatry.2021.2099 (PMC8482304; doi:10.1001/jamapsychiatry.2021.2099)
Supplement: Supplement 8. — Nonauthor Collaborators [file jamapsychiatry-e212099-s008.pdf]

Supplemental Online Content: Nonauthor Collaborators

*Indicates required information. Only first name, last name, and suffix will appear in PubMed.

*Group Name(s): 23andMe Research Team, China Kadoorie Biobank Collaborative Group, Major Depressive Disorder Working Group of the Psychiatric Genomics Consortium							
***First Name and Middle Initial(s)**	***Last Name**	***Suffix (eg, Jr, III)**	Academic Degrees	Institution	Location (city, state/province, country)	Role or Contribution, eg, chair, principal investigator	Group (if more than 1 Group listed in the byline) and/or Subgroup (eg, Steering Committee)
Naomi R.	Wray			The University of Queensland	Brisbane, QLD, AU	investigator	PGC
Stephan	Ripke			Broad Institute	Cambridge, MA, US	investigator	PGC
Stephan	Ripke			Massachusetts General Hospital	Boston, MA, US	investigator	PGC
Stephan	Ripke			Universitätsmedizin Berlin Campus Charité Mitte	Berlin, DE	investigator	PGC
Manuel	Mattheisen			Aarhus University	Aarhus, DK	investigator	PGC
Manuel	Mattheisen			iPSYCH, The Lundbeck Foundation Initiative for Integrative Psychiatric Research	DK	investigator	PGC
Manuel	Mattheisen			Karolinska Institutet	Stockholm, SE	investigator	PGC
Maciej	Trzaskowski			The University of Queensland	Brisbane, QLD, AU	investigator	PGC
Enda M.	Byrne			The University of Queensland	Brisbane, QLD, AU	investigator	PGC
Abdel	Abdellaoui			Vrije Universiteit Amsterdam	Amsterdam, NL	investigator	PGC
Mark J.	Adams			University of Edinburgh	Edinburgh, GB	investigator	PGC
Esben	Agerbo			Aarhus University	Aarhus, DK	investigator	PGC
Esben	Agerbo			iPSYCH, The Lundbeck Foundation Initiative for Integrative Psychiatric Research	DK	investigator	PGC
Tracy M.	Air			University of Adelaide	Adelaide, SA, AU	investigator	PGC
Till F. M.	Andlauer			Max Planck Institute of Psychiatry	Munich, DE	investigator	PGC
Till F. M.	Andlauer			Munich Cluster for Systems Neurology (SyNergy)	Munich, DE	investigator	PGC
Silviu-Alin	Bacanu			Virginia Commonwealth University	Richmond, VA, US	investigator	PGC
Marie	Bækvad-Hansen			iPSYCH, The Lundbeck Foundation Initiative for Integrative Psychiatric Research	DK	investigator	PGC

*Indicates required information. Only first name, last name, and suffix will appear in PubMed.

*First Name and Middle Initial(s)	*Last Name	*Suffix (eg, Jr, III)	Academic Degrees	Institution	Location (city, state/province, country)	Role or Contribution, eg, chair, principal investigator	Group (if more than 1 Group listed in the byline) and/or Subgroup (eg, Steering Committee)
Marie	Bækvad-Hansen			Statens Serum Institut	Copenhagen, DK	investigator	PGC
Aartjan T. F.	Beekman			Vrije Universiteit Medical Center and GGZ inGeest	Amsterdam, NL	investigator	PGC
Tim B.	Bigdeli			Virginia Institute for Psychiatric and Behavior Genetics	Richmond, VA, US	investigator	PGC
Elisabeth B.	Binder			Emory University School of Medicine	Atlanta, GA, US	investigator	PGC
Elisabeth B.	Binder			Max Planck Institute of Psychiatry	Munich, DE	investigator	PGC
Julien	Bryois			Karolinska Institutet	Stockholm, SE	investigator	PGC
Henriette N.	Buttenschøn			Aarhus University	Aarhus, DK	investigator	PGC
Henriette N.	Buttenschøn			iPSYCH, The Lundbeck Foundation Initiative for Integrative Psychiatric Research	DK	investigator	PGC
Jonas	Bybjerg-Grauholm			iPSYCH, The Lundbeck Foundation Initiative for Integrative Psychiatric Research	DK	investigator	PGC
Jonas	Bybjerg-Grauholm			Statens Serum Institut	Copenhagen, DK	investigator	PGC
Na	Cai			European Bioinformatics Institute (EMBL-EBI)	Cambridge, GB	investigator	PGC
Na	Cai			Wellcome Trust Sanger Institute	Cambridge, GB	investigator	PGC
Enrique	Castelao			University Hospital of Lausanne	Prilly, Vaud, CH	investigator	PGC
Jane Hvarregaard	Christensen			Aarhus University	Aarhus, DK	investigator	PGC
Jane Hvarregaard	Christensen			iPSYCH, The Lundbeck Foundation Initiative for Integrative Psychiatric Research	DK	investigator	PGC
Toni-Kim	Clarke			University of Edinburgh	Edinburgh, GB	investigator	PGC
Jonathan R. I.	Coleman			King's College London	London, GB	investigator	PGC

Supplemental Online Content: Nonauthor Collaborators

*Indicates required information. Only first name, last name, and suffix will appear in PubMed.

*First Name and Middle Initial(s)	*Last Name	*Suffix (eg, Jr, III)	Academic Degrees	Institution	Location (city, state/province, country)	Role or Contribution, eg, chair, principal investigator	Group (if more than 1 Group listed in the byline) and/or Subgroup (eg, Steering Committee)
Lucía	Colodro-Conde			QIMR Berghofer Medical Research Institute	Herston, QLD, AU	investigator	PGC
Hilary	Coon			University of Utah	Salt Lake City, US	investigator	PGC
Baptiste	Couvy-Duchesne			The University of Queensland	Saint Lucia, QLD, AU	investigator	PGC
Nick	Craddock			Cardiff University	Cardiff, GB	investigator	PGC
Gregory E.	Crawford			Duke University	Durham, NC, US	investigator	PGC
Gail	Davies			University of Edinburgh	Edinburgh, GB	investigator	PGC
Ian J.	Deary			University of Edinburgh	Edinburgh, GB	investigator	PGC
Franziska	Degenhardt			University of Bonn	Bonn, DE	investigator	PGC
Eske M.	Derks			QIMR Berghofer Medical Research Institute	Herston, QLD, AU	investigator	PGC
Nese	Direk			Dokuz Eylul University School Of Medicine	Izmir, TR	investigator	PGC
Nese	Direk			Erasmus MC	Rotterdam, Zuid-Holland, NL	investigator	PGC
Conor V.	Dolan			Vrije Universiteit Amsterdam	Amsterdam, NL	investigator	PGC
Erin C.	Dunn			Broad Institute	Cambridge, MA, US	investigator	PGC
Erin C.	Dunn			Massachusetts General Hospital	Boston, MA, US	investigator	PGC
Thalia C.	Eley			King's College London	London, GB	investigator	PGC
Valentina	Escott-Price			Cardiff University	Cardiff, GB	investigator	PGC
Farnush Farhadi Has	Kiadeh			University of British Columbia	Vancouver, BC, CA	investigator	PGC
Hilary K.	Finucane			Harvard T.H. Chan School of Public Health	Boston, MA, US	investigator	PGC
Hilary K.	Finucane			Massachusetts Institute of Technology	Cambridge, MA, US	investigator	PGC
Jerome C.	Foo			Heidelberg University	Mannheim, Baden-Württemberg, DE	investigator	PGC
Andreas J.	Forstner			University of Basel	Basel, CH	investigator	PGC
Andreas J.	Forstner			University of Bonn	Bonn, DE	investigator	PGC

Supplemental Online Content: Nonauthor Collaborators

*Indicates required information. Only first name, last name, and suffix will appear in PubMed.

*First Name and Middle Initial(s)	*Last Name	*Suffix (eg, Jr, III)	Academic Degrees	Institution	Location (city, state/province, country)	Role or Contribution, eg, chair, principal investigator	Group (if more than 1 Group listed in the byline) and/or Subgroup (eg, Steering Committee)
Josef	Frank			Central Institute of Mental Health, Medical Faculty Mannheim, Heidelberg University	Mannheim, Baden-Württemberg, DE	investigator	PGC
Héléna A.	Gaspar			King's College London	London, GB	investigator	PGC
Michael	Gill			Trinity College Dublin	Dublin, IE	investigator	PGC
Fernando S.	Goes			Johns Hopkins University	Baltimore, MD, US	investigator	PGC
Scott D.	Gordon			QIMR Berghofer Medical Research Institute	Brisbane, QLD, AU	investigator	PGC
Jakob	Grove			Aarhus University	Aarhus, DK	investigator	PGC
Jakob	Grove			Aarhus University	Aarhus, DK	investigator	PGC
Jakob	Grove			iPSYCH, The Lundbeck Foundation Initiative for Integrative Psychiatric Research	DK	investigator	PGC
Lynsey S.	Hall			Newcastle University	Newcastle upon Tyne, GB	investigator	PGC
Lynsey S.	Hall			University of Edinburgh	Edinburgh, GB	investigator	PGC
Christine Søholm	Hansen			iPSYCH, The Lundbeck Foundation Initiative for Integrative Psychiatric Research	DK	investigator	PGC
Christine Søholm	Hansen			Statens Serum Institut	Copenhagen, DK	investigator	PGC
Thomas F.	Hansen			Department of Neurology, Rigshospitalet	Glostrup, DK	investigator	PGC
Thomas F.	Hansen			Mental Health Services Capital Region of Denmark	Copenhagen, DK	investigator	PGC
Thomas F.	Hansen			The Lundbeck Foundation Initiative for Psychiatric Research	Copenhagen, DK	investigator	PGC
Stefan	Herms			University of Basel	Basel, CH	investigator	PGC
Stefan	Herms			University of Bonn	Bonn, DE	investigator	PGC
Ian B.	Hickie			University of Sydney	Sydney, NSW, AU	investigator	PGC

*Indicates required information. Only first name, last name, and suffix will appear in PubMed.

*First Name and Middle Initial(s)	*Last Name	*Suffix (eg, Jr, III)	Academic Degrees	Institution	Location (city, state/province, country)	Role or Contribution, eg, chair, principal investigator	Group (if more than 1 Group listed in the byline) and/or Subgroup (eg, Steering Committee)
Per	Hoffmann			University of Basel	Basel, CH	investigator	PGC
Per	Hoffmann			University of Bonn	Bonn, DE	investigator	PGC
Georg	Homuth			University Medicine and Ernst Moritz Arndt University Greifswald	Greifswald, Mecklenburg-Vorpommern, DE	investigator	PGC
Carsten	Horn			F. Hoffmann-La Roche Ltd	Basel, CH	investigator	PGC
Jouke-Jan	Hottenga			Vrije Universiteit Amsterdam	Amsterdam, NL	investigator	PGC
David M.	Howard			University of Edinburgh	Edinburgh, GB	investigator	PGC
David M.	Howard			King's College London	London, GB	investigator	PGC
David M.	Hougaard			iPSYCH, The Lundbeck Foundation Initiative for Integrative Psychiatric Research	DK	investigator	PGC
David M.	Hougaard			Statens Serum Institut	Copenhagen, DK	investigator	PGC
Marcus	Ising			Max Planck Institute of Psychiatry	Munich, DE	investigator	PGC
Rick	Jansen			Vrije Universiteit Medical Center and GGZ inGeest	Amsterdam, NL	investigator	PGC
Ian	Jones			Cardiff University	Cardiff, GB	investigator	PGC
Lisa A.	Jones			University of Worcester	Worcester, GB	investigator	PGC
Eric	Jorgenson			Kaiser Permanente Northern California	Oakland, CA, US	investigator	PGC
James A.	Knowles			University of Southern California	Los Angeles, CA, US	investigator	PGC
Isaac S.	Kohane			Boston Children's Hospital	Boston, MA, US	investigator	PGC
Isaac S.	Kohane			Brigham and Women's Hospital	Boston, MA, US	investigator	PGC
Isaac S.	Kohane			Harvard Medical School	Boston, MA, US	investigator	PGC
Julia	Kraft			Universitätsmedizin Berlin Campus Charité Mitte	Berlin, DE	investigator	PGC
Warren W.	Kretzschmar			University of Oxford	Oxford, GB	investigator	PGC
Zoltán	Kutalik			Swiss Institute of Bioinformatics	Lausanne, VD, CH	investigator	PGC

*Indicates required information. Only first name, last name, and suffix will appear in PubMed.

*First Name and Middle Initial(s)	*Last Name	*Suffix (eg, Jr, III)	Academic Degrees	Institution	Location (city, state/province, country)	Role or Contribution, eg, chair, principal investigator	Group (if more than 1 Group listed in the byline) and/or Subgroup (eg, Steering Committee)
Zoltán	Kutalik			University Hospital of Lausanne	Lausanne, VD, CH	investigator	PGC
Yihan	Li			University of Oxford	Oxford, GB	investigator	PGC
Penelope A.	Lind			QIMR Berghofer Medical Research Institute	Herston, QLD, AU	investigator	PGC
Jurjen J.	Luykx			University Medical Center Utrecht, Utrecht University	Utrecht, NL	investigator	PGC
Jurjen J.	Luykx			GGNet Mental Health	Apeldoorn, NL	investigator	PGC
Donald J.	MacIntyre			NHS 24	Glasgow, GB	investigator	PGC
Donald J.	MacIntyre			University of Edinburgh	Edinburgh, GB	investigator	PGC
Dean F.	MacKinnon			Johns Hopkins University	Baltimore, MD, US	investigator	PGC
Robert M.	Maier			The University of Queensland	Brisbane, QLD, AU	investigator	PGC
Wolfgang	Maier			University of Bonn	Bonn, DE	investigator	PGC
Jonathan	Marchini			University of Oxford	Oxford, GB	investigator	PGC
Hamdi	Mbarek			Vrije Universiteit Amsterdam	Amsterdam, NL	investigator	PGC
Patrick	McGrath			Columbia University College of Physicians and Surgeons	New York, NY, US	investigator	PGC
Peter	McGuffin			King's College London	London, GB	investigator	PGC
Sarah E.	Medland			QIMR Berghofer Medical Research Institute	Herston, QLD, AU	investigator	PGC
Divya	Mehta			Queensland University of Technology	Brisbane, QLD, AU	investigator	PGC
Divya	Mehta			The University of Queensland	Brisbane, QLD, AU	investigator	PGC
Christel M.	Middeldorp			Children's Health Queensland Hospital and Health Service	South Brisbane, QLD, AU	investigator	PGC
Christel M.	Middeldorp			University of Queensland	Brisbane, QLD, AU	investigator	PGC
Christel M.	Middeldorp			Vrije Universiteit Amsterdam	Amsterdam, NL	investigator	PGC
Evelin	Mihailov			University of Tartu	Tartu, EE	investigator	PGC
Yuri	Milaneschi			Vrije Universiteit Medical Center and GGZ inGeest	Amsterdam, NL	investigator	PGC
Lili	Milani			University of Tartu	Tartu, EE	investigator	PGC
Francis M.	Mondimore			Johns Hopkins University	Baltimore, MD, US	investigator	PGC

*Indicates required information. Only first name, last name, and suffix will appear in PubMed.

*First Name and Middle Initial(s)	*Last Name	*Suffix (eg, Jr, III)	Academic Degrees	Institution	Location (city, state/province, country)	Role or Contribution, eg, chair, principal investigator	Group (if more than 1 Group listed in the byline) and/or Subgroup (eg, Steering Committee)
Grant W.	Montgomery			The University of Queensland	Brisbane, QLD, AU	investigator	PGC
Sara	Mostafavi			University of British Columbia	Vancouver, BC, CA	investigator	PGC
Niamh	Mullins			King's College London	London, GB	investigator	PGC
Matthias	Nauck			University Medicine Greifswald	Greifswald, Mecklenburg-Vorpommern, DE	investigator	PGC
Bernard	Ng			University of British Columbia	Vancouver, BC, CA	investigator	PGC
Michel G.	Nivard			Vrije Universiteit Amsterdam	Amsterdam, NL	investigator	PGC
Dale R.	Nyholt			Queensland University of Technology	Brisbane, QLD, AU	investigator	PGC
Paul F.	O'Reilly			King's College London	London, GB	investigator	PGC
Hogni	Oskarsson			Humus	Reykjavik, IS	investigator	PGC
Michael J.	Owen			Cardiff University	Cardiff, GB	investigator	PGC
Jodie N.	Painter			QIMR Berghofer Medical Research Institute	Herston, QLD, AU	investigator	PGC
Carsten Bøcker	Pedersen			Aarhus University	Aarhus, DK	investigator	PGC
Carsten Bøcker	Pedersen			iPSYCH, The Lundbeck Foundation Initiative for Integrative Psychiatric Research	DK	investigator	PGC
Marianne Giørtz	Pedersen			Aarhus University	Aarhus, DK	investigator	PGC
Marianne Giørtz	Pedersen			iPSYCH, The Lundbeck Foundation Initiative for Integrative Psychiatric Research	DK	investigator	PGC
Roseann E.	Peterson			Virginia Commonwealth University	Richmond, VA, US	investigator	PGC
Erik	Pettersson			Karolinska Institutet	Stockholm, SE	investigator	PGC
Wouter J.	Peyrot			Vrije Universiteit Medical Center and GGZ inGeest	Amsterdam, NL	investigator	PGC
Giorgio	Pistis			University Hospital of Lausanne	Prilly, Vaud, CH	investigator	PGC
Danielle	Posthuma			Vrije Universiteit Amsterdam	Amsterdam, NL	investigator	PGC
Jorge A.	Quiroz			Solid Biosciences	Boston, MA, US	investigator	PGC

Supplemental Online Content: Nonauthor Collaborators

*Indicates required information. Only first name, last name, and suffix will appear in PubMed.

*First Name and Middle Initial(s)	*Last Name	*Suffix (eg, Jr, III)	Academic Degrees	Institution	Location (city, state/province, country)	Role or Contribution, eg, chair, principal investigator	Group (if more than 1 Group listed in the byline) and/or Subgroup (eg, Steering Committee)
Per	Qvist			Aarhus University	Aarhus, DK	investigator	PGC
Per	Qvist			iPSYCH, The Lundbeck Foundation Initiative for Integrative Psychiatric Research	DK	investigator	PGC
John P.	Rice			Washington University in Saint Louis School of Medicine	Saint Louis, MO, US	investigator	PGC
Brien P.	Riley			Virginia Commonwealth University	Richmond, VA, US	investigator	PGC
Margarita	Rivera			King's College London	London, GB	investigator	PGC
Margarita	Rivera			University of Granada	Granada, ES	investigator	PGC
Saira Saeed	Mirza			Erasmus MC	Rotterdam, Zuid-Holland, NL	investigator	PGC
Robert	Schoevers			University of Groningen, University Medical Center Groningen	Groningen, NL	investigator	PGC
Eva C.	Schulte			Medical Center of the University of Munich, Campus Innenstadt	Munich, DE	investigator	PGC
Ling	Shen			Kaiser Permanente Northern California	Oakland, CA, US	investigator	PGC
Jianxin	Shi			National Cancer Institute	Bethesda, MD, US	investigator	PGC
Stanley I.	Shyn			Kaiser Permanente Washington	Seattle, WA, US	investigator	PGC
Engilbert	Sigurdsson			University of Iceland	Reykjavik, IS	investigator	PGC
Grant C. B.	Sinnamon			James Cook University	Townsville, QLD, AU	investigator	PGC
Johannes H.	Smit			Vrije Universiteit Medical Center and GGZ inGeest	Amsterdam, NL	investigator	PGC
Daniel J.	Smith			University of Glasgow	Glasgow, GB	investigator	PGC
Hreinn	Stefansson			deCODE Genetics / Amgen	Reykjavik, IS	investigator	PGC
Stacy	Steinberg			deCODE Genetics / Amgen	Reykjavik, IS	investigator	PGC

Supplemental Online Content: Nonauthor Collaborators

*Indicates required information. Only first name, last name, and suffix will appear in PubMed.

*First Name and Middle Initial(s)	*Last Name	*Suffix (eg, Jr, III)	Academic Degrees	Institution	Location (city, state/province, country)	Role or Contribution, eg, chair, principal investigator	Group (if more than 1 Group listed in the byline) and/or Subgroup (eg, Steering Committee)
Fabian	Streit			Central Institute of Mental Health, Medical Faculty Mannheim, Heidelberg University	Mannheim, Baden-Württemberg, DE	investigator	PGC
Jana	Strohmaier			Central Institute of Mental Health, Medical Faculty Mannheim, Heidelberg University	Mannheim, Baden-Württemberg, DE	investigator	PGC
Katherine E.	Tansey			Cardiff University	Cardiff, GB	investigator	PGC
Henning	Teismann			University of Münster	Münster, Nordrhein-Westfalen, DE	investigator	PGC
Alexander	Teumer			University Medicine Greifswald	Greifswald, Mecklenburg-Vorpommern, DE	investigator	PGC
Wesley	Thompson			iPSYCH, The Lundbeck Foundation Initiative for Integrative Psychiatric Research	DK	investigator	PGC
Wesley	Thompson			Mental Health Services Capital Region of Denmark	Copenhagen, DK	investigator	PGC
Wesley	Thompson			Oslo University Hospital	Oslo, NO	investigator	PGC
Wesley	Thompson			University of California, San Diego	San Diego, CA, US	investigator	PGC
Pippa A.	Thompson			University of Edinburgh	Edinburgh, GB	investigator	PGC
Thorgeir E.	Thorgeirsson			deCODE Genetics / Amgen	Reykjavik, IS	investigator	PGC
Matthew	Traylor			University of Cambridge	Cambridge, GB	investigator	PGC
Jens	Treutlein			Central Institute of Mental Health, Medical Faculty Mannheim, Heidelberg University	Mannheim, Baden-Württemberg, DE	investigator	PGC
Vassily	Trubetskoy			Universitätsmedizin Berlin Campus Charité Mitte	Berlin, DE	investigator	PGC

*Indicates required information. Only first name, last name, and suffix will appear in PubMed.

*First Name and Middle Initial(s)	*Last Name	*Suffix (eg, Jr, III)	Academic Degrees	Institution	Location (city, state/province, country)	Role or Contribution, eg, chair, principal investigator	Group (if more than 1 Group listed in the byline) and/or Subgroup (eg, Steering Committee)
André G.	Uitterlinden			Erasmus MC	Rotterdam, Zuid-Holland, NL	investigator	PGC
Daniel	Umbricht			F. Hoffmann-La Roche Ltd	Basel, CH	investigator	PGC
Sandra	Van der Auwera			University Medicine Greifswald	Greifswald, Mecklenburg-Vorpommern, DE	investigator	PGC
Albert M.	van Hemert			Leiden University Medical Center	Leiden, NL	investigator	PGC
Alexander	Viktorin			Karolinska Institutet	Stockholm, SE	investigator	PGC
Peter M.	Visscher			The University of Queensland	Brisbane, QLD, AU	investigator	PGC
Yunpeng	Wang			iPSYCH, The Lundbeck Foundation Initiative for Integrative Psychiatric Research	DK	investigator	PGC
Yunpeng	Wang			Mental Health Services Capital Region of Denmark	Copenhagen, DK	investigator	PGC
Yunpeng	Wang			Oslo University Hospital	Oslo, NO	investigator	PGC
Bradley T.	Webb			Virginia Commonwealth University	Richmond, VA, US	investigator	PGC
Shantel Marie	Weinsheimer			iPSYCH, The Lundbeck Foundation Initiative for Integrative Psychiatric Research	DK	investigator	PGC
Shantel Marie	Weinsheimer			Mental Health Services Capital Region of Denmark	Copenhagen, DK	investigator	PGC
Jürgen	Wellmann			University of Münster	Münster, Nordrhein-Westfalen, DE	investigator	PGC
Gonneke	Willemsen			Vrije Universiteit Amsterdam	Amsterdam, NL	investigator	PGC
Stephanie H.	Witt			Central Institute of Mental Health, Medical Faculty Mannheim, Heidelberg University	Mannheim, Baden-Württemberg, DE	investigator	PGC
Yang	Wu			The University of Queensland	Brisbane, QLD, AU	investigator	PGC

*Indicates required information. Only first name, last name, and suffix will appear in PubMed.

*First Name and Middle Initial(s)	*Last Name	*Suffix (eg, Jr, III)	Academic Degrees	Institution	Location (city, state/province, country)	Role or Contribution, eg, chair, principal investigator	Group (if more than 1 Group listed in the byline) and/or Subgroup (eg, Steering Committee)
Hualin S.	Xi			Pfizer Global Research and Development	Cambridge, MA, US	investigator	PGC
Jian	Yang			The University of Queensland	Brisbane, QLD, AU	investigator	PGC
Futao	Zhang			The University of Queensland	Brisbane, QLD, AU	investigator	PGC
Volker	Arolt			University of Münster	Münster, Nordrhein-Westfalen, DE	investigator	PGC
Bernhard T	Baune			University of Adelaide	Adelaide, SA, AU	investigator	PGC
Klaus	Berger			University of Münster	Münster, Nordrhein-Westfalen, DE	investigator	PGC
Dorret I.	Boomsma			Vrije Universiteit Amsterdam	Amsterdam, NL	investigator	PGC
Sven	Cichon			Research Center Juelich	Juelich, DE	investigator	PGC
Sven	Cichon			University of Basel	Basel, CH	investigator	PGC
Sven	Cichon			University of Bonn	Bonn, DE	investigator	PGC
Udo	Dannlowski			University of Münster	Münster, Nordrhein-Westfalen, DE	investigator	PGC
EJC	de Geus			Vrije Universiteit Amsterdam	Amsterdam, NL	investigator	PGC
EJC	de Geus			Vrije Universiteit Medical Center	Amsterdam, NL	investigator	PGC
J. Raymond	DePaulo			Johns Hopkins University	Baltimore, US	investigator	PGC
Enrico	Domenici			Università degli Studi di Trento	Trento, IT	investigator	PGC
Katharina	Domschke			Faculty of Medicine, University of Freiburg	Freiburg, DE	investigator	PGC
Tõnu	Esko			Broad Institute	Cambridge, MA, US	investigator	PGC
Tõnu	Esko			University of Tartu	Tartu, EE	investigator	PGC
Hans J.	Grabe			University Medicine Greifswald	Greifswald, DE	investigator	PGC
Steven P.	Hamilton			Kaiser Permanente Northern California	San Francisco, US	investigator	PGC
Caroline	Hayward			University of Edinburgh	Edinburgh, GB	investigator	PGC
Andrew C.	Heath			Washington University in Saint Louis School of Medicine	Saint Louis, MO, US	investigator	PGC

*Indicates required information. Only first name, last name, and suffix will appear in PubMed.

*First Name and Middle Initial(s)	*Last Name	*Suffix (eg, Jr, III)	Academic Degrees	Institution	Location (city, state/province, country)	Role or Contribution, eg, chair, principal investigator	Group (if more than 1 Group listed in the byline) and/or Subgroup (eg, Steering Committee)
Kenneth S.	Kendler			Virginia Commonwealth University	Richmond, VA, US	investigator	PGC
Stefan	Kloiber			Centre for Addiction and Mental Health	Toronto, ON, CA	investigator	PGC
Stefan	Kloiber			Max Planck Institute of Psychiatry	Munich, DE	investigator	PGC
Stefan	Kloiber			University of Toronto	Toronto, ON, CA	investigator	PGC
Glyn	Lewis			University College London	London, GB	investigator	PGC
Qingqin S.	Li			Janssen Research and Development, LLC	Titusville, NJ, US	investigator	PGC
Susanne	Lucae			Max Planck Institute of Psychiatry	Munich, DE	investigator	PGC
Pamela AF	Madden			Washington University in Saint Louis School of Medicine	Saint Louis, MO, US	investigator	PGC
Patrik K.	Magnusson			Karolinska Institutet	Stockholm, SE	investigator	PGC
Nicholas G.	Martin			QIMR Berghofer Medical Research Institute	Brisbane, QLD, AU	investigator	PGC
Andrew M.	McIntosh			University of Edinburgh	Edinburgh, GB	chair	PGC
Andres	Metspalu			University of Tartu	Tartu, EE	investigator	PGC
Ole	Mors			Aarhus University Hospital, Risskov	Aarhus, DK	investigator	PGC
Ole	Mors			iPSYCH, The Lundbeck Foundation Initiative for Integrative Psychiatric Research	DK	investigator	PGC
Preben Bo	Mortensen			Aarhus University	Aarhus, DK	investigator	PGC
Preben Bo	Mortensen			iPSYCH, The Lundbeck Foundation Initiative for Integrative Psychiatric Research	DK	investigator	PGC
Bertram	Müller-Myhsok			Max Planck Institute of Psychiatry	Munich, DE	investigator	PGC

*Indicates required information. Only first name, last name, and suffix will appear in PubMed.

*First Name and Middle Initial(s)	*Last Name	*Suffix (eg, Jr, III)	Academic Degrees	Institution	Location (city, state/province, country)	Role or Contribution, eg, chair, principal investigator	Group (if more than 1 Group listed in the byline) and/or Subgroup (eg, Steering Committee)
Bertram	Müller-Myhsok			Munich Cluster for Systems Neurology (SyNergy)	Munich, DE	investigator	PGC
Bertram	Müller-Myhsok			University of Liverpool	Liverpool, GB	investigator	PGC
Merete	Nordentoft			Copenhagen Universtity Hospital	Copenhagen, DK	investigator	PGC
Merete	Nordentoft			iPSYCH, The Lundbeck Foundation Initiative for Integrative Psychiatric Research	DK	investigator	PGC
Markus M.	Nöthen			University of Bonn	Bonn, DE	investigator	PGC
Michael C.	O'Donovan			Cardiff University	Cardiff, GB	investigator	PGC
Sara A.	Paciga			Pfizer Global Research and Development	Groton, CT, US	investigator	PGC
Nancy L.	Pedersen			Karolinska Institutet	Stockholm, SE	investigator	PGC
Brenda WJH	Penninx			Vrije Universiteit Medical Center and GGZ inGeest	Amsterdam, NL	investigator	PGC
Roy H.	Perlis			Harvard Medical School	Boston, MA, US	investigator	PGC
Roy H.	Perlis			Massachusetts General Hospital	Boston, MA, US	investigator	PGC
David J.	Porteous			University of Edinburgh	Edinburgh, GB	investigator	PGC
James B.	Potash			University of Iowa	Iowa City, IA, US	investigator	PGC
Martin	Preisig			University Hospital of Lausanne	Prilly, Vaud, CH	investigator	PGC
Marcella	Rietschel			Central Institute of Mental Health, Medical Faculty Mannheim, Heidelberg University	Mannheim, Baden-Württemberg, DE	investigator	PGC
Catherine	Schaefer			Kaiser Permanente Northern California	Oakland, CA, US	investigator	PGC
Thomas G.	Schulze			Central Institute of Mental Health, Medical Faculty Mannheim, Heidelberg University	Mannheim, Baden-Württemberg, DE	investigator	PGC

*Indicates required information. Only first name, last name, and suffix will appear in PubMed.

*First Name and Middle Initial(s)	*Last Name	*Suffix (eg, Jr, III)	Academic Degrees	Institution	Location (city, state/province, country)	Role or Contribution, eg, chair, principal investigator	Group (if more than 1 Group listed in the byline) and/or Subgroup (eg, Steering Committee)
Thomas G.	Schulze			Johns Hopkins University	Baltimore, MD, US	investigator	PGC
Thomas G.	Schulze			Medical Center of the University of Munich, Campus Innenstadt	Munich, DE	investigator	PGC
Thomas G.	Schulze			NIMH Division of Intramural Research Programs	Bethesda, MD, US	investigator	PGC
Thomas G.	Schulze			University Medical Center Göttingen	Goettingen, DE	investigator	PGC
Jordan W.	Smoller			Broad Institute	Cambridge, MA, US	investigator	PGC
Jordan W.	Smoller			Massachusetts General Hospital	Boston, MA, US	investigator	PGC
Kari	Stefansson			deCODE Genetics / Amgen	Reykjavik, IS	investigator	PGC
Kari	Stefansson			University of Iceland	Reykjavik, IS	investigator	PGC
Henning	Tiemeier			Erasmus MC	Rotterdam, Zuid-Holland, NL	investigator	PGC
Rudolf	Uher			Dalhousie University	Halifax, NS, CA	investigator	PGC
Henry	Völzke			University Medicine Greifswald	Greifswald, Mecklenburg-Vorpommern, DE	investigator	PGC
Myrna M.	Weissman			Columbia University College of Physicians and Surgeons	New York, NY, US	investigator	PGC
Myrna M.	Weissman			New York State Psychiatric Institute	New York, NY, US	investigator	PGC
Thomas	Werge			iPSYCH, The Lundbeck Foundation Initiative for Integrative Psychiatric Research	DK	investigator	PGC
Thomas	Werge			Mental Health Services Capital Region of Denmark	Copenhagen, DK	investigator	PGC
Thomas	Werge			University of Copenhagen	Copenhagen, DK	investigator	PGC
Cathryn M.	Lewis			King's College London	London, GB	chair	PGC
Douglas F.	Levinson			Stanford University	Stanford, CA, US	investigator	PGC
Gerome	Breen			King's College London	London, GB	investigator	PGC

Supplemental Online Content: Nonauthor Collaborators

*Indicates required information. Only first name, last name, and suffix will appear in PubMed.

*First Name and Middle Initial(s)	*Last Name	*Suffix (eg, Jr, III)	Academic Degrees	Institution	Location (city, state/province, country)	Role or Contribution, eg, chair, principal investigator	Group (if more than 1 Group listed in the byline) and/or Subgroup (eg, Steering Committee)
Anders D.	Børglum			Aarhus University	Aarhus, DK	investigator	PGC
Anders D.	Børglum			iPSYCH, The Lundbeck Foundation Initiative for Integrative Psychiatric Research	DK	investigator	PGC
Patrick F.	Sullivan			Karolinska Institutet	Stockholm, SE	investigator	PGC
Patrick F.	Sullivan			University of North Carolina at Chapel Hill	Chapel Hill, NC, US	investigator	PGC
Michelle	Agee			23andMe, Inc			23andMe
Stella	Aslibekyan			23andMe, Inc			23andMe
Adam	Auton			23andMe, Inc			23andMe
Elizabeth	Babalola			23andMe, Inc			23andMe
Robert K.	Bell			23andMe, Inc			23andMe
Jessica	Bielenberg			23andMe, Inc			23andMe
Katarzyna	Bryc			23andMe, Inc			23andMe
Emily	Bullis			23andMe, Inc			23andMe
Briana	Cameron			23andMe, Inc			23andMe
Daniella	Coker			23andMe, Inc			23andMe
Gabriel	Cuellar Partida			23andMe, Inc			23andMe
Devika	Dhamija			23andMe, Inc			23andMe
Sayantan	Das			23andMe, Inc			23andMe
Sarah L.	Elson			23andMe, Inc			23andMe
Teresa	Filshtein			23andMe, Inc			23andMe
Kipper	Fletez-Brant			23andMe, Inc			23andMe
Pierre	Fontanillas			23andMe, Inc			23andMe
Will	Freyman			23andMe, Inc			23andMe
Pooja M.	Gandhi			23andMe, Inc			23andMe
Karl	Heilbron			23andMe, Inc			23andMe
Barry	Hicks			23andMe, Inc			23andMe
David A.	Hinds			23andMe, Inc			23andMe

Supplemental Online Content: Nonauthor Collaborators

*Indicates required information. Only first name, last name, and suffix will appear in PubMed.

*First Name and Middle Initial(s)	*Last Name	*Suffix (eg, Jr, III)	Academic Degrees	Institution	Location (city, state/province, country)	Role or Contribution, eg, chair, principal investigator	Group (if more than 1 Group listed in the byline) and/or Subgroup (eg, Steering Committee)
Karen E.	Huber			23andMe, Inc			23andMe
Ethan M.	Jewett			23andMe, Inc			23andMe
Yunxuan	Jiang			23andMe, Inc			23andMe
Aaron	Kleinman			23andMe, Inc			23andMe
Katelyn	Kukar			23andMe, Inc			23andMe
Vanessa	Lane			23andMe, Inc			23andMe
Keng-Han	Lin			23andMe, Inc			23andMe
Maya	Lowe			23andMe, Inc			23andMe
Marie K.	Luff			23andMe, Inc			23andMe
Jennifer C.	McCreight			23andMe, Inc			23andMe
Matthew H.	McIntyre			23andMe, Inc			23andMe
Kimberly F.	McManus			23andMe, Inc			23andMe
Steven J.	Micheletti			23andMe, Inc			23andMe
Meghan E.	Moreno			23andMe, Inc			23andMe
Joanna L.	Mountain			23andMe, Inc			23andMe
Sahar V.	Mozaffari			23andMe, Inc			23andMe
Priyanka	Nandakumar			23andMe, Inc			23andMe
Elizabeth S.	Noblin			23andMe, Inc			23andMe
Jared	O'Connell			23andMe, Inc			23andMe
Aaron A.	Petrakovitz			23andMe, Inc			23andMe
G. David	Poznik			23andMe, Inc			23andMe
Morgan	Schumacher			23andMe, Inc			23andMe
Anjali J.	Shastri			23andMe, Inc			23andMe
Janie F.	Shelton			23andMe, Inc			23andMe
Jingchunzi	Shi			23andMe, Inc			23andMe
Suyash	Shringarpure			23andMe, Inc			23andMe
Chao	Tian			23andMe, Inc			23andMe
Vinh	Tran			23andMe, Inc			23andMe
Joyce Y.	Tung			23andMe, Inc			23andMe
Xin	Wang			23andMe, Inc			23andMe

Supplemental Online Content: Nonauthor Collaborators

*Indicates required information. Only first name, last name, and suffix will appear in PubMed.

***First Name and Middle Initial(s)**	***Last Name**	***Suffix (eg, Jr, III)**	Academic Degrees	Institution	Location (city, state/province, country)	Role or Contribution, eg, chair, principal investigator	Group (if more than 1 Group listed in the byline) and/or Subgroup (eg, Steering Committee)
Wei	Wang			23andMe, Inc			23andMe
Catherine H.	Weldon			23andMe, Inc			23andMe
Peter	Wilton			23andMe, Inc			23andMe
Daniel	Avery			University of Oxford	Oxford, GB		CKB
Derrick	Bennett			University of Oxford	Oxford, GB		CKB
Zheng	Bian			Chinese Academy of Medical Sciences	Beijing, CN		CKB
Ruth	Boxall			University of Oxford	Oxford, GB		CKB
Fiona	Bragg			University of Oxford	Oxford, GB		CKB
Ka Hung	Chan			University of Oxford	Oxford, GB		CKB
Liang	Chang			Centre for Disease Control and Prevention	Qingdao Province, Shandong, CN		CKB
Yumei	Chang			University of Oxford	Oxford, GB		CKB
Biyun	Chen			Centre for Disease Control and Prevention	Hunan Province, Changsha, CN		CKB
Jinyan	Chen			Centre for Disease Control and Prevention	Hainan Province, Haikou, CN		CKB
Junshi	Chen			China National Center for Food Safety Risk Assessment	Beijing, CN	Steering Committee	CKB
Naying	Chen			Centre for Disease Control and Prevention	Guangxi Province, Nanning, China		CKB
Ningyu	Chen			Liuzhou Centre for Disease Control and Prevention	Guangxi Province, Liuzhou, CN		CKB
Xiaofang	Chen			Pengzhou Centre for Disease Control and Prevention	Sichuan Province, Pengzhou, CN		CKB
Xiaofang	Chen			Centre for Disease Control and Prevention	Sichuan Province, Chengdu, CN		CKB
Yiping	Chen			University of Oxford	Oxford, GB		CKB
Zhengming	Chen			University of Oxford	Oxford, GB	co-Principal Investigato	CKB
Liang	Cheng			Centre for Disease Control and Prevention	Qingdao Province, Shandong, CN		CKB

Supplemental Online Content: Nonauthor Collaborators

*Indicates required information. Only first name, last name, and suffix will appear in PubMed.

*First Name and Middle Initial(s)	*Last Name	*Suffix (eg, Jr, III)	Academic Degrees	Institution	Location (city, state/province, country)	Role or Contribution, eg, chair, principal investigator	Group (if more than 1 Group listed in the byline) and/or Subgroup (eg, Steering Committee)
Johnathan	Clarke			University of Oxford	Oxford, GB		CKB
Robert	Clarke			University of Oxford	Oxford, GB	Steering Committee	CKB
Rory	Collins			University of Oxford	Oxford, GB	Steering Committee	CKB
Caixia	Dong			Centre for Disease Control and Prevention	Gansu Province, Lanzhou, CN		CKB
Huaidong	Du			University of Oxford	Oxford, GB		CKB
Ranran	Du			Centre for Disease Control and Prevention	Qingdao Province, Shandong, China		CKB
Zammy	Fairhurst-Hunter			University of Oxford	Oxford, GB		CKB
Lei	Fan			Centre for Disease Control and Prevention	Qingdao Province, Shandong, CN		CKB
Shixian	Feng			Centre for Disease Control and Prevention	Qingdao Province, Shandong, China		CKB
Zhongxi	Fu			Centre for Disease Control and Prevention	Hunan Province, Changsha, CN		CKB
Wei	Gan			University of Oxford	Oxford, GB		CKB
Ruqin	Gao			Centre for Disease Control and Prevention	Qingdao Province, Shandong, CN		CKB
Yulian	Gao			Xuixian Centre for Disease Control and Prevention	Henan Province, Huixian, China		CKB
Pengfei	Ge			Centre for Disease Control and Prevention	Gansu Province, Lanzhou, CN		CKB
Simon	Gilbert			University of Oxford	Oxford, GB		CKB
Weiwei	Gong			Centre for Disease Control and Prevention	Zhejiang Province, Hangzhou, CN		CKB
Qijun	Gu			Tongxiang Centre for Disease Control and Prevention	Zhejiang Province, Tongxiang, CN		CKB
Yu	Guo			Chinese Academy of Medical Sciences	Beijing, CN	Steering Committee	CKB
Zhendong	Guo			Meilan Centre for Disease Control and Prevention	Hainan Province, Haikou, CN		CKB

*Indicates required information. Only first name, last name, and suffix will appear in PubMed.

***First Name and Middle Initial(s)**	***Last Name**	***Suffix (eg, Jr, III)**	Academic Degrees	Institution	Location (city, state/province, country)	Role or Contribution, eg, chair, principal investigator	Group (if more than 1 Group listed in the byline) and/or Subgroup (eg, Steering Committee)
Ziyan	Guo			Nangang Centre for Disease Control and Prevention	Heilongjiang Province, Harbin, CN		CKB
Alex	Hacker			University of Oxford	Oxford, GB		CKB
Xiao	Han			Chinese Academy of Medical Sciences	Beijing, CN		CKB
Parisa	Hariri			University of Oxford	Oxford, GB		CKB
Pan	He			Xuixian Centre for Disease Control and Prevention	Henan Province, Huixian, CN		CKB
Tianyou	He			Xuixian Centre for Disease Control and Prevention	Henan Province, Huixian, CN		CKB
Mike	Hill			University of Oxford	Oxford, GB		CKB
Michael	Holmes			University of Oxford	Oxford, GB		CKB
Michael	Holmes			Oxford University Hospital	Oxford, GB		CKB
Can	Hou			Chinese Academy of Medical Sciences	Beijing, CN		CKB
Wei	Hou			Licang Centre for Disease Control and Prevention	Qingdao Province, Shandong, CN		CKB
Chen	Hu			Xuixian Centre for Disease Control and Prevention	Henan Province, Huixian, CN		CKB
Ruying	Hu			Centre for Disease Control and Prevention	Zhejiang Province, Hangzhou, CN		CKB
Ximin	Hu			Centre for Disease Control and Prevention	Hainan Province, Haikou, CN		CKB
Yihe	Hu			Suzhou Centre for Disease Control and Prevention	Jiangsu Province, Suzhou, CN		CKB
Hua	Hua			Centre for Disease Control and Prevention	Qingdao Province, Shandong, CN		CKB
Yujie	Hua			Suzhou Centre for Disease Control and Prevention	Jiangsu Province, Suzhou, CN		CKB
Yuelong	Huang			Centre for Disease Control and Prevention	Hunan Province, Changsha, CN		CKB

Supplemental Online Content: Nonauthor Collaborators

*Indicates required information. Only first name, last name, and suffix will appear in PubMed.

*First Name and Middle Initial(s)	*Last Name	*Suffix (eg, Jr, III)	Academic Degrees	Institution	Location (city, state/province, country)	Role or Contribution, eg, chair, principal investigator	Group (if more than 1 Group listed in the byline) and/or Subgroup (eg, Steering Committee)
Pek Kei	Im			University of Oxford	Oxford, GB		CKB
Andri	Iona			University of Oxford	Oxford, GB		CKB
Qilian	Jiang			Liuzhou Centre for Disease Control and Prevention	Guangxi Province, Liuzhou, CN		CKB
Jianrong	Jin			Suzhou Centre for Disease Control and Prevention	Jiangsu Province, Suzhou, CN		CKB
Maria	Kakkoura			University of Oxford	Oxford, GB		CKB
Quan	Kang			Nangang Centre for Disease Control and Prevention	Heilongjiang Province, Harbin, CN		CKB
Christiana	Kartsonaki			University of Oxford	Oxford, GB		CKB
Rene	Kerosi			University of Oxford	Oxford, GB		CKB
Ling	Kong			University of Oxford	Oxford, GB		CKB
Jian	Lan			Liuzhou Centre for Disease Control and Prevention	Guangxi Province, Liuzhou, CN		CKB
Garry	Lancaster			University of Oxford	Oxford, GB		CKB
Feifei	Li			Centre for Disease Control and Prevention	Qingdao Province, Shandong, CN		CKB
Huimei	Li			Meilan Centre for Disease Control and Prevention	Hainan Province, Haikou, CN		CKB
Jianguo	Li			Pengzhou Centre for Disease Control and Prevention	Sichuan Province, Pengzhou, CN		CKB
Liming	Li			Peking University Health Science Center	Beijing, CN	co-Principal Investigato	CKB
Mingqiang	Li			Liuzhou Centre for Disease Control and Prevention	Guangxi Province, Liuzhou, CN		CKB
Shanpeng	LI			Centre for Disease Control and Prevention	Qingdao Province, Shandong, CN		CKB
Yanjie	Li			Nangang Centre for Disease Control and Prevention	Heilongjiang Province, Harbin, CN		CKB
Yilei	Li			Meilan Centre for Disease Control and Prevention	Hainan Province, Haikou, CN		CKB

*Indicates required information. Only first name, last name, and suffix will appear in PubMed.

***First Name and Middle Initial(s)**	***Last Name**	***Suffix (eg, Jr, III)**	Academic Degrees	Institution	Location (city, state/province, country)	Role or Contribution, eg, chair, principal investigator	Group (if more than 1 Group listed in the byline) and/or Subgroup (eg, Steering Committee)
Zhongxiao	Li			Maiji Centre for Disease Control and Prevention	Gansu Province, Tianshui, CN		CKB
Kuang	Lin			University of Oxford	Oxford, GB		CKB
Lingli	Lingli			Tongxiang Centre for Disease Control and Prevention	Zhejiang Province, Tongxiang, CN		CKB
Chao	Liu			Chinese Academy of Medical Sciences	Beijing, CN		CKB
Depei	Liu			Chinese Academy of Medical Sciences	Beijing, CN		CKB
Duo	Liu			Centre for Disease Control and Prevention	Guangxi Province, Nanning, China		CKB
Fang	Liu			Suzhou Centre for Disease Control and Prevention	Jiangsu Province, Suzhou, CN		CKB
Huilin	Liu			Centre for Disease Control and Prevention	Hunan Province, Changsha, CN		CKB
Jiaqiu	Liu			Pengzhou Centre for Disease Control and Prevention	Sichuan Province, Pengzhou, CN		CKB
Jingchao	Liu			Suzhou Centre for Disease Control and Prevention	Jiangsu Province, Suzhou, CN		CKB
Yongmei	Liu			Centre for Disease Control and Prevention	Qingdao Province, Shandong, CN		CKB
Yun	Liu			Liuzhou Centre for Disease Control and Prevention	Guangxi Province, Liuzhou, CN		CKB
Huajun	Long			Liuyang Centre for Disease Control and Prevention	Hunan Province, Liuyang, CN		CKB
Yan	Lu			Suzhou Centre for Disease Control and Prevention	Jiangsu Province, Suzhou, CN		CKB
Guojin	Luo			Pengzhou Centre for Disease Control and Prevention	Sichuan Province, Pengzhou, CN		CKB
Jun	Lv			Peking University Health Science Center	Beijing, CN	Steering Committee	CKB

*Indicates required information. Only first name, last name, and suffix will appear in PubMed.

*First Name and Middle Initial(s)	*Last Name	*Suffix (eg, Jr, III)	Academic Degrees	Institution	Location (city, state/province, country)	Role or Contribution, eg, chair, principal investigator	Group (if more than 1 Group listed in the byline) and/or Subgroup (eg, Steering Committee)
Silu	Lv			Licang Centre for Disease Control and Prevention	Qingdao Province, Shandong, CN		CKB
Liangcai	Ma			Suzhou Centre for Disease Control and Prevention	Jiangsu Province, Suzhou, CN		CKB
Enke	Mao			Maiji Centre for Disease Control and Prevention	Gansu Province, Tianshui, CN		CKB
John	McDonnell			University of Oxford	Oxford, GB		CKB
Fanwen	Meng			Liuzhou Centre for Disease Control and Prevention	Guangxi Province, Liuzhou, CN		CKB
Jinhuai	Meng			Liuzhou Centre for Disease Control and Prevention	Guangxi Province, Liuzhou, CN		CKB
Iona	Millwood			University of Oxford	Oxford, GB		CKB
Qunhua	Nie			University of Oxford	Oxford, GB		CKB
Feng	Ning			Centre for Disease Control and Prevention	Qingdao Province, Shandong, CN		CKB
Dongxia	Pan			Tongxiang Centre for Disease Control and Prevention	Zhejiang Province, Tongxiang, CN		CKB
Rong	Pan			Liuzhou Centre for Disease Control and Prevention	Guangxi Province, Liuzhou, CN		CKB
Zengchang	Pang			Centre for Disease Control and Prevention	Qingdao Province, Shandong, CN		CKB
Pei	Pei			Chinese Academy of Medical Sciences	Beijing, CN		CKB
Richard	Peto			University of Oxford	Oxford, GB	Steering Committee	CKB
Alfred	Pozarickij			University of Oxford	Oxford, GB		CKB
Yijian	Qian			Tongxiang Centre for Disease Control and Prevention	Zhejiang Province, Tongxiang, CN		CKB
Yulu	Qin			Liuzhou Centre for Disease Control and Prevention	Guangxi Province, Liuzhou, CN		CKB

Supplemental Online Content: Nonauthor Collaborators

*Indicates required information. Only first name, last name, and suffix will appear in PubMed.

***First Name and Middle Initial(s)**	***Last Name**	***Suffix (eg, Jr, III)**	Academic Degrees	Institution	Location (city, state/province, country)	Role or Contribution, eg, chair, principal investigator	Group (if more than 1 Group listed in the byline) and/or Subgroup (eg, Steering Committee)
Chan	Qu			Liuyang Centre for Disease Control and Prevention	Hunan Province, Liuyang, CN		CKB
Xiaolan	Ren			Centre for Disease Control and Prevention	Gansu Province, Lanzhou, CN		CKB
Paul	Ryder			University of Oxford	Oxford, GB		CKB
Sam	Sansome			University of Oxford	Oxford, GB		CKB
Dan	Schmidt			University of Oxford	Oxford, GB		CKB
Paul	Sherliker			University of Oxford	Oxford, GB		CKB
Rajani	Sohoni			University of Oxford	Oxford, GB		CKB
Becky	Stevens			University of Oxford	Oxford, GB		CKB
Jian	Su			Centre for Disease Control and Prevention	Jiangsu Province, Nanjing, CN		CKB
Huarong	Sun			Xuixian Centre for Disease Control and Prevention	Henan Province, Huixian, CN		CKB
Qiang	Sun			Pengzhou Centre for Disease Control and Prevention	Sichuan Province, Pengzhou, CN		CKB
Xiaohui	Sun			Centre for Disease Control and Prevention	Qingdao Province, Shandong, CN		CKB
Aiyu	Tang			Suzhou Centre for Disease Control and Prevention	Jiangsu Province, Suzhou, CN		CKB
Zhenzhu	Tang			Centre for Disease Control and Prevention	Guangxi Province, Nanning, CN		CKB
Ran	Tao			Centre for Disease Control and Prevention	Jiangsu Province, Nanjing, CN		CKB
Xiaocao	Tian			Centre for Disease Control and Prevention	Qingdao Province, Shandong, CN		CKB
Iain	Turnbull			University of Oxford	Oxford, GB		CKB
Robin	Walters			University of Oxford	Oxford, GB	Steering Committee	CKB
Meng	Wan			Centre for Disease Control and Prevention	Zhejiang Province, Hangzhou, CN		CKB

*Indicates required information. Only first name, last name, and suffix will appear in PubMed.

***First Name and Middle Initial(s)**	***Last Name**	***Suffix (eg, Jr, III)**	Academic Degrees	Institution	Location (city, state/province, country)	Role or Contribution, eg, chair, principal investigator	Group (if more than 1 Group listed in the byline) and/or Subgroup (eg, Steering Committee)
Chunmei	Wang			Tongxiang Centre for Disease Control and Prevention	Zhejiang Province, Tongxiang, CN		CKB
Chen	Wang			Chinese Academy of Medical Sciences	Beijing, CN	Steering Committee	CKB
Hao	Wang			Centre for Disease Control and Prevention	Zhejiang Province, Hangzhou, CN		CKB
Junzheng	Wang			Licang Centre for Disease Control and Prevention	Qingdao Province, Shandong, CN		CKB
Lin	Wang			University of Oxford	Oxford, GB		CKB
Ping	Wang			Liuzhou Centre for Disease Control and Prevention	Guangxi Province, Liuzhou, CN		CKB
Tao	Wang			Maiji Centre for Disease Control and Prevention	Gansu Province, Tianshui, CN		CKB
Shaojie	Wang			Centre for Disease Control and Prevention	Qingdao Province, Shandong, CN		CKB
Sisi	Wang			Liuzhou Centre for Disease Control and Prevention	Guangxi Province, Liuzhou, CN		CKB
Xiaohuan	Wang			Centre for Disease Control and Prevention	Hainan Province, Haikou, CN		CKB
Liuping	Wei			Liuzhou Centre for Disease Control and Prevention	Guangxi Province, Liuzhou, CN		CKB
Min	Weng			Meilan Centre for Disease Control and Prevention	Hainan Province, Haikou, CN		CKB
Neil	Wright			University of Oxford	Oxford, GB		CKB
Ming	Wu			Centre for Disease Control and Prevention	Jiangsu Province, Nanjing, CN		CKB
Xianping	Wu			Centre for Disease Control and Prevention	Sichuan Province, Chengdu, CN		CKB
Shukuan	Wu			Meilan Centre for Disease Control and Prevention	Hainan Province, Haikou, CN		CKB

Supplemental Online Content: Nonauthor Collaborators

*Indicates required information. Only first name, last name, and suffix will appear in PubMed.

*First Name and Middle Initial(s)	*Last Name	*Suffix (eg, Jr, III)	Academic Degrees	Institution	Location (city, state/province, country)	Role or Contribution, eg, chair, principal investigator	Group (if more than 1 Group listed in the byline) and/or Subgroup (eg, Steering Committee)
Kaixu	Xie			Tongxiang Centre for Disease Control and Prevention	Zhejiang Province, Tongxiang, CN		CKB
Qiaohua	Xu			Centre for Disease Control and Prevention	Hunan Province, Changsha, CN		CKB
Qinai	Xu			Nangang Centre for Disease Control and Prevention	Heilongjiang Province, Harbin, CN		CKB
Xin	Xu			Liuyang Centre for Disease Control and Prevention	Hunan Province, Liuyang, CN		CKB
Shichun	Yan			Centre for Disease Control and Prevention	Heilongjiang Province, Harbin, CN		CKB
Ling	Yang			University of Oxford	Oxford, GB		CKB
Xiaoming	Yang			University of Oxford	Oxford, GB		CKB
Jie	Yang			Centre for Disease Control and Prevention	Jiangsu Province, Nanjing, CN		CKB
Pang	Yao			University of Oxford	Oxford, GB		CKB
Li	Yin			Centre for Disease Control and Prevention	Hunan Province, Changsha, CN		CKB
Bo	Yu			Nangang Centre for Disease Control and Prevention	Heilongjiang Province, Harbin, CN		CKB
Canqing	Yu			Chinese Academy of Medical Sciences	Beijing, CN		CKB
Min	Yu			Centre for Disease Control and Prevention	Zhejiang Province, Hangzhou, CN		CKB
Yaoming	Zhai			Centre for Disease Control and Prevention	Qingdao Province, Shandong, CN		CKB
Hao	Zhang			Liuyang Centre for Disease Control and Prevention	Hunan Province, Liuyang, CN		CKB
Hui	Zhang			Maiji Centre for Disease Control and Prevention	Gansu Province, Tianshui, CN		CKB
Jun	Zhang			Suzhou Centre for Disease Control and Prevention	Jiangsu Province, Suzhou, CN		CKB

Supplemental Online Content: Nonauthor Collaborators

*Indicates required information. Only first name, last name, and suffix will appear in PubMed.

***First Name and Middle Initial(s)**	***Last Name**	***Suffix (eg, Jr, III)**	Academic Degrees	Institution	Location (city, state/province, country)	Role or Contribution, eg, chair, principal investigator	Group (if more than 1 Group listed in the byline) and/or Subgroup (eg, Steering Committee)
Libo	Zhang			Liuyang Centre for Disease Control and Prevention	Hunan Province, Liuyang, CN		CKB
Ningmei	Zhang			Centre for Disease Control and Prevention	Sichuan Province, Chengdu, CN		CKB
Xi	Zhang			Maiji Centre for Disease Control and Prevention	Gansu Province, Tianshui, CN		CKB
Xiaoyi	Zhang			Tongxiang Centre for Disease Control and Prevention	Zhejiang Province, Tongxiang, CN		CKB
Xukui	Zhang			Huixian Centre for Disease Control and Prevention	Henan Province, Huixian, CN		CKB
Xunfu	Zhong			Pengzhou Centre for Disease Control and Prevention	Sichuan Province, Pengzhou, CN		CKB
Ding Zhang	Zhou			Centre for Disease Control and Prevention	Qingdao Province, Shandong, CN		CKB
Gang	Zhou			Centre for Disease Control and Prevention	Qingdao Province, Shandong, CN		CKB
Jinyi	Zhou			Centre for Disease Control and Prevention	Jiangsu Province, Nanjing, CN		CKB
Liyuan	Zhou			Liuzhou Centre for Disease Control and Prevention	Guangxi Province, Liuzhou, CN		CKB
Weiwei	Zhou			Centre for Disease Control and Prevention	Sichuan Province, Chengdu, CN		CKB
Xue	Zhou			Centre for Disease Control and Prevention	Heilongjiang Province, Harbin, CN		CKB
Yonglin	Zhou			Centre for Disease Control and Prevention	Jiangsu Province, Nanjing, CN		CKB
Mingyuan	Zou			Centre for Disease Control and Prevention	Heilongjiang Province, Harbin, CN		CKB
